# *Borrelia burgdorferi* tolerates alteration to P66 porin function in a murine infectivity model

**DOI:** 10.3389/fcimb.2024.1528456

**Published:** 2025-01-21

**Authors:** Christa H. Fierros, Marie-Line Faucillion, Beth L. Hahn, Phillip Anderson, Mari Bonde, Julie R. Kessler, Matthew C. Surdel, Kyler S. Crawford, Yan Gao, Jieqing Zhu, Sven Bergström, Jenifer Coburn

**Affiliations:** ^1^ Department of Microbiology and Immunology, Medical College of Wisconsin, Milwaukee, WI, United States; ^2^ Department of Molecular Biology, Umeå University, Umea, Sweden; ^3^ Department of Medicine, Medical College of Wisconsin, Milwaukee, WI, United States; ^4^ Department of Biochemistry, Medical College of Wisconsin, Milwaukee, WI, United States; ^5^ Division of Biostatistics, Medical College of Wisconsin, Milwaukee, WI, United States; ^6^ Department of Biochemistry, Medical College of Wisconsin, Versiti Blood Research Institute, Milwaukee, WI, United States

**Keywords:** P66, porin, *Borrelia burgdorferi*, Lyme disease, bacterial pathogenesis, tickborne pathogen

## Abstract

*Borrelia burgdorferi* exists in a complex enzootic life cycle requiring differential gene regulation. P66, a porin and adhesin, is upregulated and essential during mammalian infection, but is not produced or required within the tick vector. We sought to determine whether the porin function of P66 is essential for infection. Vancomycin treatment of *B. burgdorferi* cultures was used to screen for P66 porin function and found to generate spontaneous mutations in *p66* (*bb0603*). Three novel, spontaneous, missense P66 mutants (G175V, T176M, and G584R) were re-created by site-directed mutagenesis in an infectious strain background and tested for infectivity in mice by ID_50_ experiments. Two of the three mutants retained infectivity comparable to the isogenic control, suggesting that *B. burgdorferi* can tolerate alteration to P66 porin function during infection. The third mutant exhibited highly attenuated infectivity and produced low levels of P66 protein. Interestingly, four isolates that were recovered for *p66* sequencing from mouse tissues revealed novel secondary point mutations in genomic *p66*. However, these secondary mutations did not rescue P66 porin function. New structural modeling of P66 is presented and consistent with these experimental results. This is the first work to assess the contribution of P66 porin function to *B. burgdorferi* pathogenesis.

## 1 Introduction

Lyme disease is the most common arthropod-borne infection in the United States and is found across the Northern hemisphere (CDC, 2024; Stanek et al., 2012; Nelson et al., 2015). The number of cases has risen annually with recent estimates exceeding 300,000 cases in the United States (Nelson et al., 2015; Schwartz et al., 2021). Lyme disease is caused by infection with certain species of gram-negative bacteria of the *Borrelia* genus (Burgdorfer et al., 1982); *B*. *burgdorferi* is the predominant Lyme disease-causing species in North America. *B. burgdorferi* is an obligate parasite maintained in an enzootic life cycle between certain *Ixodes* tick species and vertebrate hosts (Burgdorfer et al., 1982; Anderson, 1988; Pal and Fikrig, 2003). Humans are incidental hosts and acquire a *B. burgdorferi* infection through a tick bite. In the absence of a visible erythema migrans lesion, accurate timely diagnosis is hampered by nonspecific early symptoms. Furthermore, serological methods are the primary laboratory tests for Lyme disease, which depend on the production of antibodies that only occurs weeks after infection (Lantos et al., 2021). Untreated Lyme disease can progress to arthritis in the weight-bearing joints, carditis, and neuritis of the peripheral and central nervous system (Radolf et al., 2010; Steere et al., 2016). These symptoms may not resolve with antibiotic treatment and persist long-term. The geographic range of the tick vector is expanding (Khatchikian et al., 2015; Eisen et al., 2016), which will likely lead to Lyme disease cases in new areas. Although several groups are working toward the development of new vaccines for Lyme disease (Gomes-Solecki et al., 2020; Guibinga et al., 2020; Izac et al., 2020; Kamp et al., 2020; Stafford et al., 2020; O’Bier et al., 2021; Oliveira et al., 2021; Trentelman et al., 2021), none have been approved for human use. Taken together, Lyme disease is a growing public health threat.

P66 is a 66 kDa protein chromosomally encoded by *bb0603* that resides in the outer membrane of all characterized *Borrelia* species (Bárcena-Uribarri et al., 2010). In *B. burgdorferi*, the protein is produced during mammalian infection but not while inside the tick vector (Coleman and Benach, 1987; Cugini et al., 2003; Ristow et al., 2012). P66 is recognized by human sera and was among the earliest identified antigenic proteins of *B. burgdorferi* (Coleman and Benach, 1987; Dressler et al., 1993). Considering the antigenicity of P66, interactions of P66 with host components have been explored. These investigations included the generation of the *p66* KO4 strain in which approximately the middle third of the *p66* coding sequence was replaced with a kanamycin resistance cassette, resulting in no detection of protein or fragments thereof (Coburn and Cugini, 2003; Ristow et al., 2012). This strain is abbreviated as *Δp66* throughout this paper. *B. burgdorferi Δp66* is noninfectious in wild-type (WT), TLR2^-/-^ (Ristow et al., 2012), MyD88^-/-^ (Ristow et al., 2012), RAG^-/-^ mice (Curtis and Coburn, unpub.), neutrophil depleted (Curtis et al., 2017), macrophage depleted (Ristow et al., 2015), or dendritic cell depleted (Ristow et al., 2015) mice. Similarly, *Δp66* and WT are equally sensitive to mCRAMP (antimicrobial peptide in mouse skin) and to complement *in vitro* (Ristow et al., 2015; Curtis et al., 2017). The *Δp66* mutant has similar characteristics to WT organisms when assessed *in vitro* for membrane integrity, chemotactic response, and nutrient acquisition (Curtis et al., 2017). In other studies, P66 was shown to be an adhesin that binds to β_3_ chain (α_IIb_β_3_ and α_v_β_3_) integrins (Coburn et al., 1999; Cugini et al., 2003) which is dependent on 181-187 amino acid (aa) in the mature protein (Ristow et al., 2015). This integrin-binding function facilitates extravasation and dissemination of *B. burgdorferi* during infection but is not essential for infectivity (Ristow et al., 2012; Kumar et al., 2015; Ristow et al., 2015). Jointly, these results suggest that a function of P66 essential during mammalian infection has not yet been defined.

Although the P66 structure remains unsolved, it is a porin (Barbour et al., 1984; Coleman and Benach, 1987; Skare et al., 1997; Pinne et al., 2006). The literature definition of a porin has evolved over time. Broadly speaking, porins are β-barrel proteins that span the outer membrane of gram-negative bacteria and permit the movement of molecules across the membrane. General diffusion, substrate-specific (with ligand-binding), and active transport proteins have all been termed porins (Nikaido, 1992). General (or classic) porins are defined as water-filled channels in the outer membrane that facilitate the passive diffusion of small (<600 Da) hydrophilic molecules (Galdiero et al., 2012). In general, porins form β-barrels in the outer membrane, have a cleavable ~21 aa signal sequence, have either 16 or 18 transmembrane β-strands, and diameters ranging from 6-15 Å (Galdiero et al., 2012). Although monomeric porins are documented, porins typically oligomerize as trimers wherein each monomer forms a channel. Porins also tend to have a loop within the channel that constricts the center of the pore and affects permeability. A variety of bacterial porins are known to facilitate passage of cations or anions, various phosphate molecules (Modi et al., 2015), sugars (Meyer et al., 1997; Wang et al., 1997; Andersen et al., 1998; Adewoye and Worobec, 2000), nucleosides (Nieweg and Bremer, 1997), alginate (Rehm, 1996; Rehman and Rehm, 2013), amides and urea (Mills et al., 1997), and other molecules. P66 was experimentally determined to exhibit porin activity in planar lipid bilayer and proteoliposome experiments (Skare et al., 1997).

Only two other channel-forming proteins aside from P66 have been molecularly characterized in *Borrelia* spirochetes: DipA and the P13 porin family. In a black lipid bilayer assay, a protein of 38 kDa, designated DipA (for dicarboxylate-specific porin A), with an average single-channel conductance of 50 picoSiemans (pS) in 1 M KCl was characterized. The DipA porin is selective for anions such as oxaloacetate, 2-oxoglutarate, and citrate. Data suggest that DipA does not form a general diffusion pore, but a porin with a binding site specific for dicarboxylates which play important key roles in the deficient metabolic and biosynthetic pathways of *Borrelia* species (Thein et al., 2008; Thein et al., 2012).

The P13 porin BB0034 belongs to the paralogous family 48. PF48 has eight additional plasmid-encoded genes or pseudo-genes (Fraser et al., 1997; Noppa et al., 2001; Pinne et al., 2006). The pore-forming activity of P13 was first described in 2002 using protein purified by FPLC from an outer membrane protein preparation. In a black lipid bilayer assay, it displayed an average single channel conductance of 3.5 nanoSiemans (nS) (Östberg et al., 2002). However, in a subsequent study using a different purification method, the single channel conductance was revised and confirmed to be 0.6 nS (Barcena-Uribarri et al., 2014). P13 is able to form channels in the borrelial outer membrane despite its small molecular mass and α-helical secondary structure (Östberg et al., 2002; Barcena-Uribarri et al., 2014). This homo-oligomeric porin possibly acts as a general diffusion channel for small molecules into *Borrelia* (Barcena-Uribarri et al., 2014).

P66 is a uniquely large porin: it is predicted to have 22 or 24 transmembrane strands, oligomerizes as a heptamer or octamer, and has an estimated diameter of ~19 Å with a central constriction of ~8 Å (Barcena-Uribarri et al., 2013; Kenedy et al., 2014). It also exhibits an unusually high channel conductance of approximately 11 nS in 1 M KCl as measured by black lipid bilayer (BLB) (Pinne et al., 2006). It is hypothesized that this large channel conductance is due to the oligomerization of P66 [~480 kDa band is observed by blue native polyacrylamide gel electrophoresis (BN-PAGE) of outer membrane fractions of *B. burgdorferi* (Curtis et al., 2022)], wherein 7 or 8 monomers with individual 1.5 nS conductance account for the 11 nS signal (Skare et al., 1997; Barcena-Uribarri et al., 2013; Ristow et al., 2015). The biological substrates and degree of selectivity of P66 remain unknown.

The aim of this study was to determine whether the porin function of P66 is essential for the infectivity of *B. burgdorferi*. Toward this end, we utilized a vancomycin-based screen for porin function, generated missense mutants with altered porin function, and assessed infectivity of those mutants in mice. We demonstrate that alteration of P66 porin function can be tolerated *in vivo*. Furthermore, structural modeling of P66 with AlphaFold (Evans et al., 2021; Jumper et al., 2021) is consistent with these experimental results.

## 2 Materials and methods

### 2.1 Bacterial strains and growth conditions

*E. coli* strains were grown in Luria Bertani (LB) medium at 30°C supplemented with 0.02% dextrose. Gentamicin was used at 10 μg/ml and ampicillin at 100 µg/ml where appropriate.

All *Borrelia* strains were cultured in Barbour-Stoenner-Kelly II [BSKII (Barbour, 1984)] medium at 33°C in the presence of antibiotics as indicated. Resistance markers were maintained with 40 μg/ml gentamicin and 200 μg/ml kanamycin.

Bacterial quantification in liquid culture was determined by darkfield microscopy using a Petroff-Hausser counting chamber. Culture density is shown as motile cells in 0.02 µl which is the volume of 25 fields of view, or 25 squares, on the counting chamber. In order to graph ‘0’ values on a logarithmic *y* axis, a value of one spirochete was added to every value prior to graphing.

### 2.2 Porin function assays

#### 2.2.1 Antibiotic susceptibility

##### 2.2.1.1 Vancomycin susceptibility

P66 porin function by BLB has been correlated to vancomycin susceptibility (Curtis et al., 2022). Vancomycin resistance corresponds to impaired P66 porin function while vancomycin susceptibility corresponds to intact P66 porin function. Strains of interest were grown to exponential phase and 5x10^6^ cells were subcultured into 5 ml BSKII with and without 1 µg/ml vancomycin. Growth was monitored by darkfield microscopy for 3 days. WT and *Δp66* strains were treated in parallel as controls.

##### 2.2.1.2 Ampicillin susceptibility

The same experiment was performed on the WT and *Δp66* strains with ampicillin at the following concentrations: 0, 0.001, 0.01, 0.1, 1, 10, 100 µg/ml. Three independent trials were conducted. Only data for the 0.1 µg/ml dose are shown as it was an intermediary dose and representative of the strains behaving identically.

#### 2.2.2 Black lipid bilayer

The isolation of the outer membrane proteins (B-fraction) of the different *Borrelia* strains was performed as described previously (Magnarelli et al., 1989; Curtis et al., 2022).

BLB experiments were performed with a Teflon chamber with two compartments separated by a thin wall and connected by a small circular hole with an area of about 0.4 mm^2^ (Benz et al., 1978). A layer of 2% (w/v) DiphPC (1,2-diphytanoyl-sn-glycerol-3-phosphatidylcholin, AVANTI Polar Lipids) in chloroform was applied around the hole and left to air dry. The two compartments were filled with 1 M KCl and the membranes were formed by painting a 1% (w/v) DiphPC solution in 90% n-decane/10% n-butanol over the hole. After bilayer formation as indicated by the area across the hole turning black, the B-fractions were diluted to a concentration of 0.1 µg/µl, then diluted again 1:10 in 1% Genapol X-080 (Fluka, Spain) and added to the KCl solution on both sides of the chamber. The current passing through the membrane was measured with a pair of Ag/AgCl electrodes with salt bridges switched in series and under a continuous 20 mV voltage. A highly sensitive current amplifier (Keithley 427, Lower Lake, CA, USA) amplified the signal by 10^10^ and the output signal was digitalized using an analogue to digital converter (USB DAQ ISO 8 ch 250 kHz 16 Bit, GOLDAMMER) and recorded and analyzed using the software DASYLab 2022 Lite (National Instruments Corp). Experiments were performed at room temperature, using B-fractions from each strain. The *Δp66* strain was excluded from this tedious process because its results have been well established in the literature (i.e. all channel activity < 4.5 nS) (Pinne et al., 2006; Curtis et al., 2022). Data from two independent B-fractions were obtained per strain. Representative data are shown.

### 2.3 High-throughput generation of missense mutants

We used a 96-well plate format to simultaneously set up 94 test cultures of 200 μl. Twenty milliliters of BSKII supplemented with 1 μg/ml vancomycin was distributed as 200 μl aliquots in the 96 well plate. WT *B. burgdorferi* B31 A3 was grown to exponential phase (>5x10^7^ cells/ml) and 4 μl inoculated into each well. Two wells were reserved for controls: WT cells without vancomycin, and medium alone for a colorimetric reference (i.e. the phenol red in the BSKII). Plates were sealed and incubated at 33°C. One week into incubation, medium remained red in the vancomycin treated wells and growth was sparse by darkfield microscopy relative to the untreated well. Plates were resealed and incubated for an additional week at which point robust growth and colorimetric changes were observed in some wells. The contents of these wells were plated in the presence of 1 μg/ml vancomycin. Single colonies representative of each vancomycin-treated well were selected and grown to exponential phase in liquid culture (with 1 μg/ml vancomycin). These isolates were further characterized.

### 2.4 Preparation of chemically competent *E. coli*

Chemically competent XL-Gold *E. coli* (purchased from Agilent) cells were prepared by growing 100 ml cultures to optical density 0.4-0.6 at 600 nm in transformation and storage protocol: 12.5 mM polyethylene glycol (8 kDa), 50 mM magnesium chloride hexahydrate, and 5% DMSO in Luria broth (Chung et al., 1989; DeCero et al., 2020). After the culture was chilled on ice for 20 min, the culture was centrifuged at 3000 RCF for 10 min at 4°C, the supernatant was discarded, and the pellet resuspended in 5 ml of chilled transformation and storage solution with 10% autoclaved glycerol. Finally, 50 μl aliquots were flash frozen in liquid nitrogen and stored at -80°C until use.

### 2.5 *E. coli* transformations

Aliquots of chemically competent XL-Gold *E. coli* cells were thawed on ice. Plasmid DNA or water was added and the cells were incubated on ice for about 8 min, heat shocked at 33°C for 40 sec, and returned to ice for 8 min. Subsequently, cells were allowed to recover in 1 ml SOC at 37°C with shaking at 225 rpm for 1 hr and plated on LB agar plates supplemented with 0.02% dextrose and selective antibiotics for 24-48 hr.

### 2.6 *B. burgdorferi* transformations

Transformations into *B. burgdorferi* strains were performed as previously described with minor modifications (Samuels, 1995; Hyde et al., 2011).

#### 2.6.1 Plasmid transformation of *p66*

For the complementation of *p66*, a 400 ml culture of *E. coli* containing the pBSV2G + *p66* clone 2-23 plasmid (*p66^cp^*) (Ristow et al., 2012) was cultured at 30°C in LB broth supplemented with 0.02% dextrose and 10 μg/ml gentamicin. This culture was used for a Plasmid Maxi-prep (Qiagen) according to manufacturer’s directions. Plasmid concentration was quantified using a NanoDrop One (ThermoScientific) and 100 μg was methylated *in vitro* by CpG MTase (NEBiolabs) and S-adenosylmethionine at 37°C for >4 hours (Chen et al., 2008). The plasmid DNA was then phenol-chloroform extracted and ethanol precipitated to concentrate the DNA for transformation. The *B. burgdorferi* P66 nonsense mutant E442* was cultured with 1 μg/ml vancomycin in BSKII and expanded to 100 ml. Genomic plasmid content of the *B. burgdorferi* culture was verified by multiplex PCR immediately prior to transformation (Bunikis et al., 2011). *B. burgdorferi* cells were prepared for transformation by pelleting and resuspending the cells in decreasing volumes of cold electroporation solution (0.272 M sucrose, 15% glycerol) (Samuels, 1995). Cells were suspended by thorough pipetting and checked for successful suspension by microscopy. Cells were split into two aliquots. Upon addition of water or plasmid DNA, the cell suspensions were further mixed and electroporated at 1800 V in ice-cold cuvettes. Cells were transferred into 5 ml of warm (~33°C) BSKII for recovery and immediately split into three tubes and denoted as three independent transformations. These cultures were allowed to recover overnight at 33°C in the absence of antibiotics. Transformations were plated the following morning. Cultures for plating were assessed by darkfield microscopy for culture density of motile cells. From these readings, 100 cells of each culture were plated in the absence of selective antibiotics. As a further control, 500 μl of the water control transformation was plated in the presence of gentamicin. Transformations with DNA (as opposed to the water control) were plated in their entirety. Plating medium was mixed at the following ratios: 12 ml of BSK 1.5X plating medium, 8 ml of 1.7% agarose (UltraPure, Invitrogen), and 208 μl of an antibiotic cocktail (stock concentrations at 1.92 mg/ml phosphomycin, 4.81 mg/ml rifampicin, 240.38 μg/ml amphotericin B; final concentrations of 20.0 µg/ml phosphomycin, 50.0 µg/ml rifampicin, 2.50 μg/ml amphotericin B). Additionally, gentamicin was used at 40 μg/ml or vancomycin at 1 μg/ml in selective plates. Plating components were mixed and ~10 ml was pipetted into the plate and allowed to solidify. Then another ~10 ml was combined with cells and pipetted on top of the already solidified layer. Plates were incubated at 33°C with 2% CO_2_ for approximately two weeks until colonies emerged. Colonies were picked into 5 ml and cultured in the presence of gentamicin until exponential phase (>5x10^7^ cells/ml) was reached. Exponential phase cultures were used for protein pellets, DNA samples, and glycerol stocks.

#### 2.6.2 Chromosomal complementation of *p66*

For transformations of chromosomal complementation (i.e. G175V, T176M, and G584R), 100 µg of plasmid DNA was digested with ApaI (37°C overnight followed by heat inactivation of 65°C for 20 min; NEBiolabs) rather than being methylated and phenol-chloroform extracted. The digested DNA was transformed into the *Δp66* background strain (Coburn and Cugini, 2003; Ristow et al., 2012).

### 2.7 Site-directed mutagenesis PCR

Site-directed mutagenesis (SDM) was achieved using the A/T-rich SDM protocol (DeCero et al., 2020). In brief, overlapping oligonucleotides were designed with the mutation of interest in both primers. Primers were designed with the QuikChange Primer Design tool from www.agilent.com to introduce single amino acid substitutions to the *p66* coding region. The pGTE-p66 plasmid (Ristow et al., 2012) was amplified with high-fidelity, HotStart Q5 polymerase (NEBiolabs) with the following cycling conditions: 98°C for 30 sec; 5 cycles of 98°C for 30 sec, 65°C (decreasing by 2°C every cycle) for 1.5 min, and 68°C for 4 min 15 sec; 15 cycles of 98°C for 30 sec, 65°C for 1.5 min, and 68°C for 4 min 15 sec; 68° for 2 min; and a final hold at 10°C. Following the PCR reaction, products were digested with DpnI to remove the parental plasmid and transformed into XL-Gold chemically competent *E. coli*. Transformants were sequence-verified prior to use in *B. burgdorferi* transformations. Primers are listed in **Supplementary Table 1.**

### 2.8 DNA extractions from bacterial cultures

Crude *B. burgdorferi* cell lysates were used as DNA templates for multiplex PCR and for PCR amplification prior to sequencing. Lysates were prepared by centrifuging 1 ml of exponential *Borrelia* culture at 11,360 x g for 8 min, discarding the supernatant, resuspending the pellet in 50 μl water, and boiling the sample at 100°C for 10 min. Lysates were stored at -20°C or -80°C.

Plasmid minipreps of *E. coli* were performed using the Wizard Plus SV Minipreps DNA Purification System (Promega) according to manufacturer’s instructions. When recovering pBSV2G-based plasmids from *Borrelia* cultures, *Borrelia* was first centrifuged at 11,360 x g for 8 min and the supernatant discarded. Resuspension of the pellet and subsequent steps were performed with the Wizard Plus SV Minipreps DNA Purification System (Promega) according to manufacturer’s instructions.

### 2.9 PCR for sequencing

The DNA concentrations of crude bacterial lysates were quantified using a NanoDrop One (ThermoScientific) and diluted to 3 ng/µl for PCR reactions. These dilutions were used as templates with primers OLCR03 (Ristow et al., 2012) and OLCR12 Rev Comp to amplify the *p66* locus. Each reaction was composed of 2 µl diluted DNA template, 1 µl of each primer (stock at 5 µM), 1 µl of 25 mM MgCl_2_, 11 µl H_2_O, and 4 µl of NEBiolabs Multiplex PCR 5X Mastermix (Bunikis et al., 2011). The thermalcycler was set with the following conditions: 95° for 2 min; 95° for 30 sec, 60° for 60 sec, and 58° for 2 min 35 sec cycled a total of 40x; 68° for 5 min; and a final 10°C hold. Following the PCR, replicate reactions were pooled and the DNA was purified using the QIAquick PCR purification kit (Qiagen) according to the manufacturer’s instructions. DNA concentrations of the products were assessed by NanoDrop One and diluted to 40 ng/µl for sequencing. Sanger sequencing was performed by Molecular Cloning Laboratories (MCLAB, San Francisco, CA) and data was analyzed by SeqMan Pro and SeqBuilder programs from DNAStar. Primers for amplification and sequencing are listed in **Supplementary Table 1.**

*B. garinii p66* was amplified as two fragments with primer sets ‘B garinii 1R’ with ‘B garinii 7F’ and ‘B garinii 6F’ with ‘OLCR 12 Rev Comp.’ Each reaction was composed of 5 µl Q5 buffer (NEBiolabs), 4 µl of 4 mM dNTP5, 2.5 µl of each primer (stock at 5 µM), 1 µl diluted DNA template (at 3 ng/µl), 2 µl of 25 mM MgCl_2_, 0.25 µl Q5 DNA polymerase (NEBiolabs), and 7.75 µl H_2_O. Both PCR fragments used the same cycling conditions: 98° for 30 sec; 98° for 10 sec, 60° for 30 sec, and 72° for 53 sec cycled a total of 35x; 72° for 2 min; and a final 10°C hold. Subsequent processing and sequencing were performed identically as the *B. burgdorferi* samples except sequencing primers differed (**Supplementary Table 1**).

### 2.10 Multiplex PCR of *B. burgdorferi* genomic plasmids

Bacterial lysates were used as DNA templates. Multiplex PCR was performed as previously described (Bunikis et al., 2011). Linear plasmid (lp), circular plasmid (cp), and T plasmid (lp5) were run in separate PCR reactions. B31 A3 WT and water templates were used as positive and negative controls (respectively) in parallel.

Both lp and cp reactions used the same cycling conditions: 95°C for 2 min; 95°C for 30 sec, 60°C for 1 min, and 68°C for 1 min cycled 40x; 68°C for 5 min; and a final hold of 25°C. Each lp and cp PCR product was combined with 6 µl of 6x xylene cyanol loading dye (1 ml of 1 M Tris-HCl pH 7.6 + 60 ml of 100% glycerol + 12 ml of 0.5 M ethylenediaminetetraacetic acid (EDTA) + 7 ml H_2_O + 30 mg of xylene cyanol). Of this, 2.5 µl was loaded on 2.5% MetaPhor agarose (Lonza) gels in tris-borate-EDTA buffer. Gels were run at ~120 V for ~2 hr, stained with ethidium bromide, and visualized (Azure Biosystems 300).

Each T plasmid reaction was composed of 2 µl 10X PCR buffer (containing 15 mM MgCl_2_, Qiagen), 1.6 µl dNTP mix (2.5 mM each of dATP, dTTP, dCTP, and dGTP), 1 µl of each primer (at 5 µM), 4 µl MgCl_2_ (25 mM, Qiagen), 1 µl DNA template, 0.1 µl HotStarTaq (5 units/µl, Qiagen), and 10.9 µl H_2_O. The T plasmid cycling reactions were as follows: 95°C for 15 min; 94°C for 30 sec, 55°C for 30 sec, and 72°C for 1 min cycled 40x; 72°C for 7 min; and a final hold of 25°C. Completed T plasmid reactions were supplemented with 3 µl of loading dye (1.2 ml of 50X Tris-acetate-EDTA (TAE) + 3.3 ml of 100% glycerol + 25 mg bromphenol blue + 25 mg xylene cyanol + 5.5 ml H_2_O) and 10 µl was loaded on 1% agarose (Bulls Eye Agarose GP2, MidSci) gels in TAE buffer. Gels were run at ~120 V for ~30 min, stained with ethidium bromide, and visualized (Azure Biosystems 300).

### 2.11 SDS-PAGE and immunoblotting

Samples for protein analysis were obtained by culturing *B. burgdorferi* strains to exponential phase (>5x10^7^ cells/ml in BSKII at 33°C) as determined by darkfield microscopy. One milliliter of culture was pelleted at 11,360 x g for 8 min, washed in 1 ml phosphate-buffered saline (PBS; 150 mM NaCl, 17 mM K_2_HPO_4_, 5 mM KH_2_PO_4_; pH 7.4), pelleted again, and diluted to 2x10^6^ cells/μl in PBS. This suspension was combined 1:1 with 2x sample buffer (made of 20 ml 0.5M Tris + 20 ml 10% SDS + 60 ml saturated sucrose + 1 ml 10% NaN_3_ + bromophenol blue to adjust color) supplemented with 2% β-mercaptoethanol for a final concentration of 1x10^6^ cells/μl. Samples were then boiled at 100°C for 5 min. Proteins were separated by running 10 μl (1x10^7^ cells) on SDS-PAGE [10% acrylamide, supplemented with 2,2,2- trichloroethanol (Acros Organics)]. Stain free gel images were obtained prior to transfer to PVDF membrane on the ChemiDoc Touch Imaging System (BioRad). Transfer was achieved by applying 1 Amp and 25 V for 30 min. PDVF membranes were blocked in 5% skim milk in tris-buffered saline (TBS) for ≥1 hour at room temperature with rocking. Membranes were washed in 1xTBS and probed simultaneously with polyclonal rabbit anti-P66 (D8713 Bleed #4 (Coburn et al., 1999) at 1:10,000 dilution) and mouse anti-flagellin (monoclonal antibody H9724 (Barbour et al., 1986) at 1:500 dilution; from T. Schwan, Rocky Mountain Laboratories) in 5% skim milk overnight at 4°C with rocking, washed in 1xTBS, and probed simultaneously with anti-rabbit IgG-HRP conjugated and anti-mouse IgG-HRP conjugated antibodies (Promega, both at 1:10,000 dilutions in 5% skim milk in TBS). Blots were developed with Clarity Western ECL substrate (Bio-Rad) and imaged by chemiluminescence on a Bio-Rad ChemiDoc. Where applicable, densitometry was performed on Image Lab 6.0 (Bio-Rad). Quantification of P66 production in the SDM mutants was derived from a single blot. All samples were subjected to the same exposure time and blotting conditions. Exposure time was selected such that no bands were supersaturated, but all were detectable.

### 2.12 Proteinase K cleavage assay

*B. burgdorferi* strains were cultured to exponential phase (>5x10^7^ cells/ml) and culture density was quantified by darkfield microscopy on a Petroff-Hausser counting chamber. Volumes calculated to contain 4.8x10^8^ cells were pelleted at 11,360 x g for 8 min and the supernatant discarded. Cells were resuspended in 800 µl of cold HBS (0.025 M HEPES, 0.15 M NaCl, 0.001 M MgCl_2_, 0.001 M MnCl_2_, 0.00025 M CaCl_2_) + 0.2% BSA + 0.1% dextrose, pelleted again, and the supernatant discarded. The cells were then resuspended in 1.6 mL of cold HBS + 0.1% dextrose (HBS+D) and divided into 6x 250 μl aliquots. These aliquots were pelleted and resuspended in 124 µl of cold HBS+D. Proteinase K (Promega) solutions were made in HBS+D and diluted to 0, 0.6, 2, 6, 20, and 60 µg/ml and 124 µl of diluted proteinase K concentrations added to the cell suspensions (final concentrations of 0, 0.3, 1, 3, 10, and 30 µg/ml Proteinase K). The reactions were incubated at room temperature for 20 min with shaking. To stop the reactions, 2 µl of 125 mM phenylmethanesulfonyl fluoride in isopropanol was added (1.0 mM final concentration) and the reactions were incubated at room temperature for 15 min with shaking. Finally, samples were again pelleted at 11,360 x g for 8 min, the supernatants discarded, and the pellets subjected to SDS-PAGE and immunoblotting. Blots were probed for flagellin and P66.

### 2.13 Minimum inhibitory concentration

MIC measurements were performed similarly to other published descriptions (Takacs et al., 2018). Strains of interest were grown to exponential phase and diluted to 2x10^5^ cells/ml in BSKII medium. A vancomycin stock was diluted to 32 µg/ml in BSKII and 2-fold serial dilutions were made in BSKII down to 0.125 µg/ml vancomycin. BSKII with diluted vancomycin was distributed as 100 µl aliquots to create a concentration gradient across a 96 well plate. Subsequently, 100 µl of the diluted cells (at 2x10^5^ cells/ml) were added to the same wells (20,000 cells per well in 200 µl) to generate a concentration range of 16 - 0.0625 µg/ml. Plates were sealed with adhesive covers and incubated at 33°C for 4 days. After incubation the MIC was determined by darkfield microscopy as the lowest concentration of vancomycin that inhibited growth. At most, two motile cells were seen across 25 fields of view in a well determined to be the MIC (≤1.0x10^5^ motile cells/ml). The control wells with 0 µg/ml vancomycin exhibited densities that were too numerous to count without dilution. Experiments were performed in technical duplicate and a minimum of three independent trials.

### 2.14 Oligomerization as visualized by BN-PAGE

The isolation of the outer membrane proteins (B-fraction) of the different *Borrelia* strains was performed as described previously (Magnarelli et al., 1989; Ristow et al., 2015; Curtis et al., 2022). BN-PAGE was performed as previously described on the B-fractions (Ristow et al., 2015; Curtis et al., 2022). Immunoblots were probed with rabbit polyclonal antibody D8713 against P66 (1/2000 dilution) followed by goat Anti-Rabbit IgG H&L (HRP) (ab205718) from Abcam (1/10,000 dilution). Bound antibodies were detected with SuperSignal West Dura Extended Duration Substrate (ThermoScientific).

### 2.15 *In silico* analyses

*Borrelia burgdorferi* P66 (Uniprot Q44644) sequence (without the 21 aa signal sequence) was used as input for AlphaFoldv2.3.1 and AlphaFold-Multimer accessed using the Medical College of Wisconsin Research Computing Center (Evans et al., 2021; Jumper et al., 2021). Additional monomeric P66 structure predictions were made of the same sequence using DeepFold accessed from the Zhang Lab server at University of Michigan (https://zhanggroup.org/DeepFold/) (Pearce et al., 2022). Multimer predictions and comparisons were performed using the GalaxyHomomer server (https://galaxy.seoklab.org/cgi-bin/submit.cgi?type=HOMOMER) from the Seok Lab at Seoul National University (Ko et al., 2012; Baek et al., 2017) initially with unbiased selection of subunits to suggest optimal conformation, and with set number of subunits for surface area and docking score comparison.

### 2.16 Mouse infection

#### 2.16.1 Infectivity of nonsense mutants

Six to eight week old female C3H/HeN mice (Charles River Laboratories) were infected subcutaneously with 1x10^5^ cells of exponential phase B31 A3 WT, B31 A3 K04 C3-14 (*Δp66*), B31 A3 E422*, B31 A3 E56*, or vehicle control (PBS + 0.2% normal mouse serum). Genomic plasmid content of the bacterial cultures was verified by multiplex PCR immediately prior to infection. Five mice per group were used. After 2 weeks, mice were euthanized by CO_2_ inhalation and tissues collected and used to inoculate 5 ml BSKII cultures in the absence of selective antibiotics (but in the presence of 20.0 µg/ml phosphomycin, 50.0 µg/ml rifampicin, and 2.50 μg/ml amphotericin B to inhibit growth of possible contaminating bacteria). Blood (~100 µl obtained by cardiac puncture), bladder, heart, a tibiotarsus (ankle), inoculation site skin (~1 cm^2^), ear, and brain tissues were harvested. Cultures were kept for up to 8 weeks and monitored for spirochete growth by darkfield microscopy. After 8 weeks, a culture was considered negative if no *B. burgdorferi* cells were observed.

#### 2.16.2 ID_50_ of missense mutants

For ID_50_ experiments, female C3H/HeN mice were approximately 5 weeks old at time of infection. *B. burgdorferi* strains of interest were grown to exponential phase, expanded into 200 ml of BSKII with selective antibiotics, profiled for genomic plasmid retention by multiplex PCR, profiled for antibiotic markers by PCR, and confirmed for P66 production (or absence) by stain free gel or immunoblot. Subsequently, cells were diluted in vehicle and used to subcutaneously inoculate groups of 5 mice across a range of doses (10^1^, 10^3^, 10^4^, 10^5^, 10^6^, 10^7^, and 10^9^ spirochetes). Two clones were tested in parallel for each mutant. Because the *Δp66* strain has been thoroughly demonstrated in the past to be noninfectious (Ristow et al., 2012; Ristow et al., 2015) and to conserve mice and reagents, only the 1x10^5^ and 1x10^9^ doses were tested for that strain. Additional infections with the WT, the *p66^cc^*, and the vehicle were performed as controls. At 4 weeks post-infection, mice were euthanized by CO_2_ inhalation and tissues collected: blood, inoculation site skin, ear, heart, bladder, tibiotarsus, and knee joint. Ear and heart samples were halved to both inoculate 5 ml of BSKII supplemented with an antibiotic cocktail (stock concentrations of 2.5 µg/ml amphotericin B, 50 µg/ml rifampicin, and 20 µg/ml phosphomycin) and to freeze for downstream qPCR assays. About 100 µl of blood was used to inoculate BSKII. For the tibiotarsus joint, left and right joints were harvested so one could be cultured and the other used in qPCR. Cultures were monitored for spirochete growth by darkfield microscopy for up to 8 weeks.

ID_50_ calculations were performed with a Probit regression model fitted to the culture data. The model was conducted by the PROC NLMIXED procedure in SAS^®^ 9.4 (SAS Institute Inc., Cary NC). The strain and log_10_-transformed dose were co-variates and the infection rate was the outcome. The ID_50_ levels were compared by using T-test statistics to determine whether the effects for the missense mutants on the dose-response relationship were equal to the corresponding effect of WT or *p66^cc^*. The raw p values were corrected for multiple comparisons by the stepdown Bonferroni method to control for the family-wise error rate.

## 3 Results

### 3.1 *B*. *burgdorferi* exhibits P66-dependent sensitivity to vancomycin, but not to ampicillin

**Figure 1:**
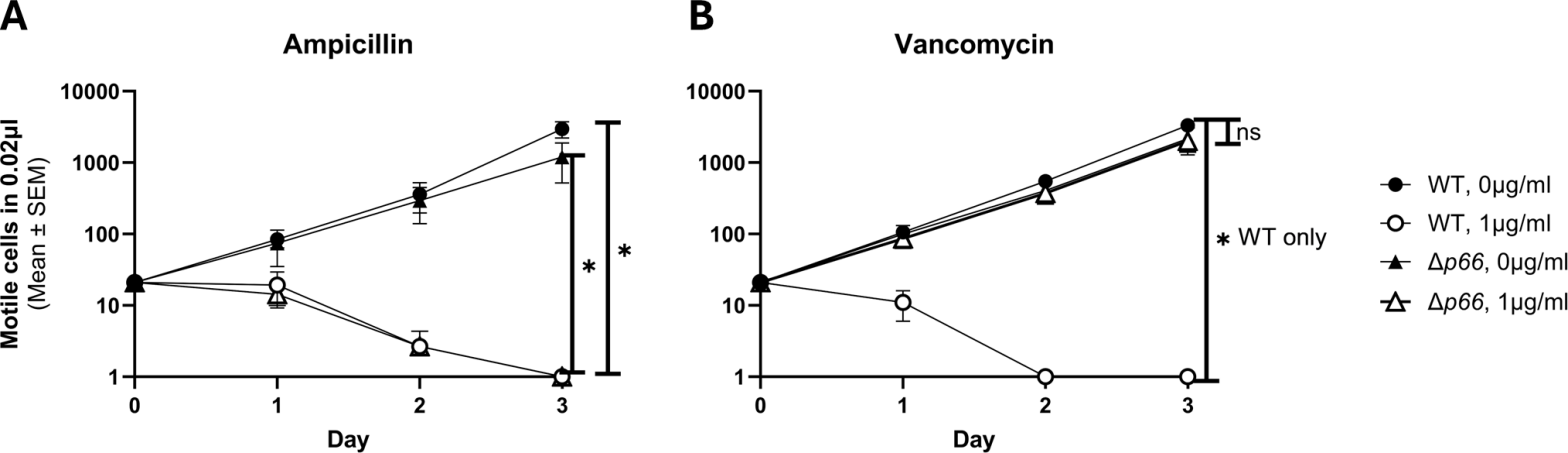
Vancomycin exhibits P66-dependent entry. B31 A3 WT and Δ*p66* strains were grown to exponential phase in BSKII and plasmid content was verified by multiplex PCR. Cells were diluted to 1x10^6^ cells/ml in the presence and absence of 0.1 µg/ml ampicillin **(A)** or 1 µg/ml vancomycin **(B)**. Culture density of motile *B. burgdorferi* was determined daily by darkfield microscopy using a Petroff-Hausser counting chamber. This experiment was performed as three independent replicates; mean and SEM are shown. GraphPad Prism 9.2.0 was used to analyze data by simple linear regression. The * denotes p <0.05 while ns (not significant) indicates p ≥0.05.

Porins facilitate passive periplasmic entry and exit of a variety of substrates through the outer membrane, including antibiotics (Galdiero et al., 2012). Antibiotic resistance can develop due to downregulating a porin, switching expression to a different porin, or mutating the porin (Pagès et al., 2008). Recently, vancomycin sensitivity was shown to correlate with P66 porin function and provides a simple and readily available way to assess porin function in the native membrane, as opposed to BLB (Curtis et al., 2022). We hypothesized that other periplasm-active antibiotics, such as ampicillin, would also exhibit P66-dependent sensitivity. To test this, *B. burgdorferi* WT and *Δp66* strains were grown in the presence of varying concentrations of antibiotics. No growth differences were observed between WT and *Δp66* strains at any tested dose of ampicillin (data for 0.1 µg/ml are shown) (**Figure 1**). Both strains were sensitive to ampicillin, implying antibiotic entry that was not P66-dependent. In contrast, when treated with 1 μg/ml vancomycin, the WT strain was sensitive while the *Δp66* strain was resistant. Both strains were sensitive to vancomycin at higher concentrations. *B. burgdorferi* exhibits P66-dependent sensitivity to vancomycin, but not to all periplasm-active antibiotics.

### 3.2 Extended vancomycin exposure *in vitro* leads to *p66* mutations

**Figure 2:**
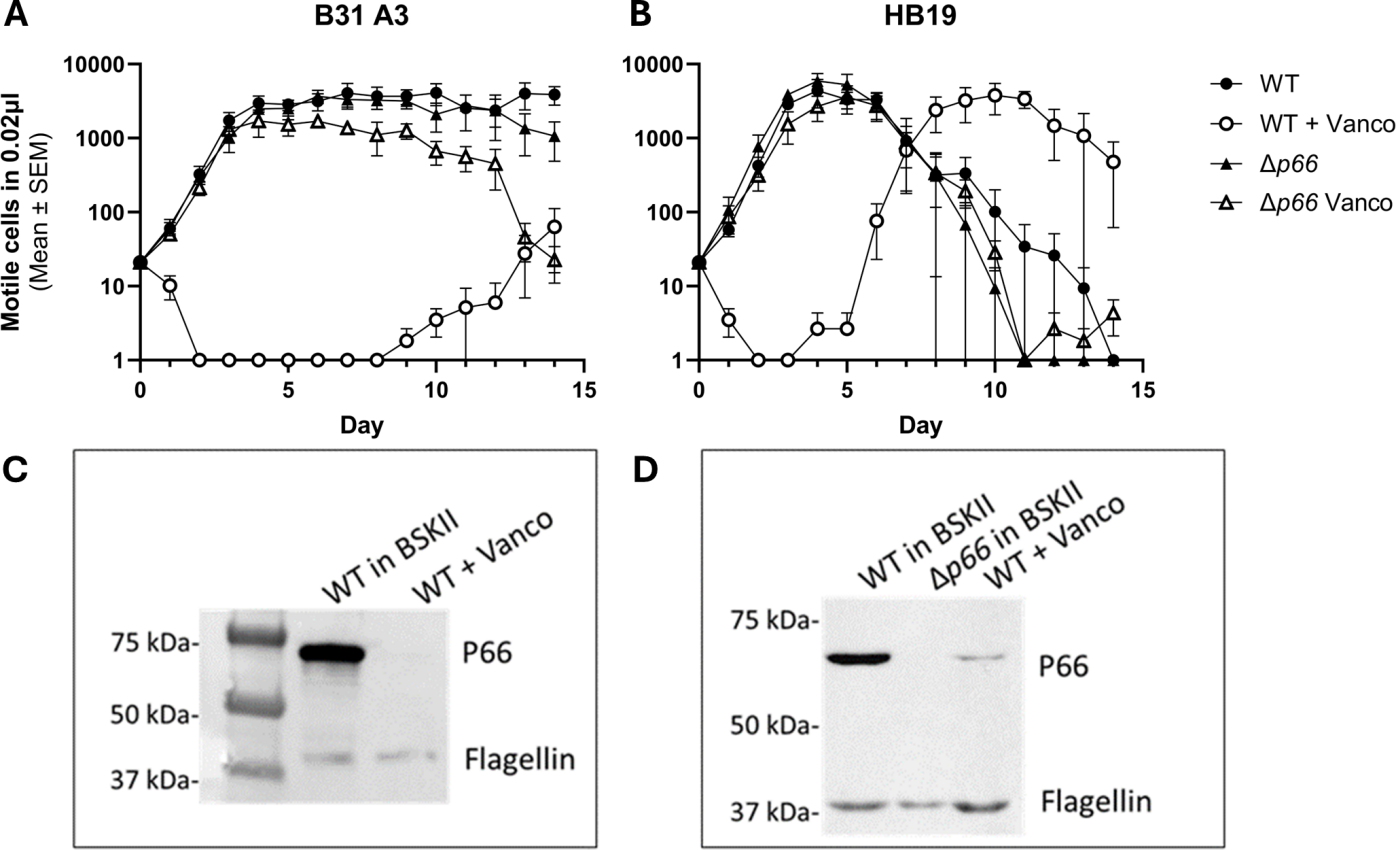
Resistant strains emerge with prolonged incubation in vancomycin. *B*. *burgdorferi* WT and Δ*p66* strains from the B31 A3 **(A)** and HB19 **(B)** strain backgrounds were cultured in BSKII medium in the presence or absence of 1 μg/ml of vancomycin for 2 weeks. Culture density readings of motile cells were taken daily by darkfield microscopy. This experiment was performed as three independent replicates; mean and SEM are shown. At the conclusion of 2 weeks, the WT cells that had been treated with vancomycin were subjected to SDS-PAGE and immunoblotting for P66 and flagellin (below corresponding growth curve) **(C, D)**. Note that after vancomycin exposure, cultures were no longer clonal.

**Figure 3:**
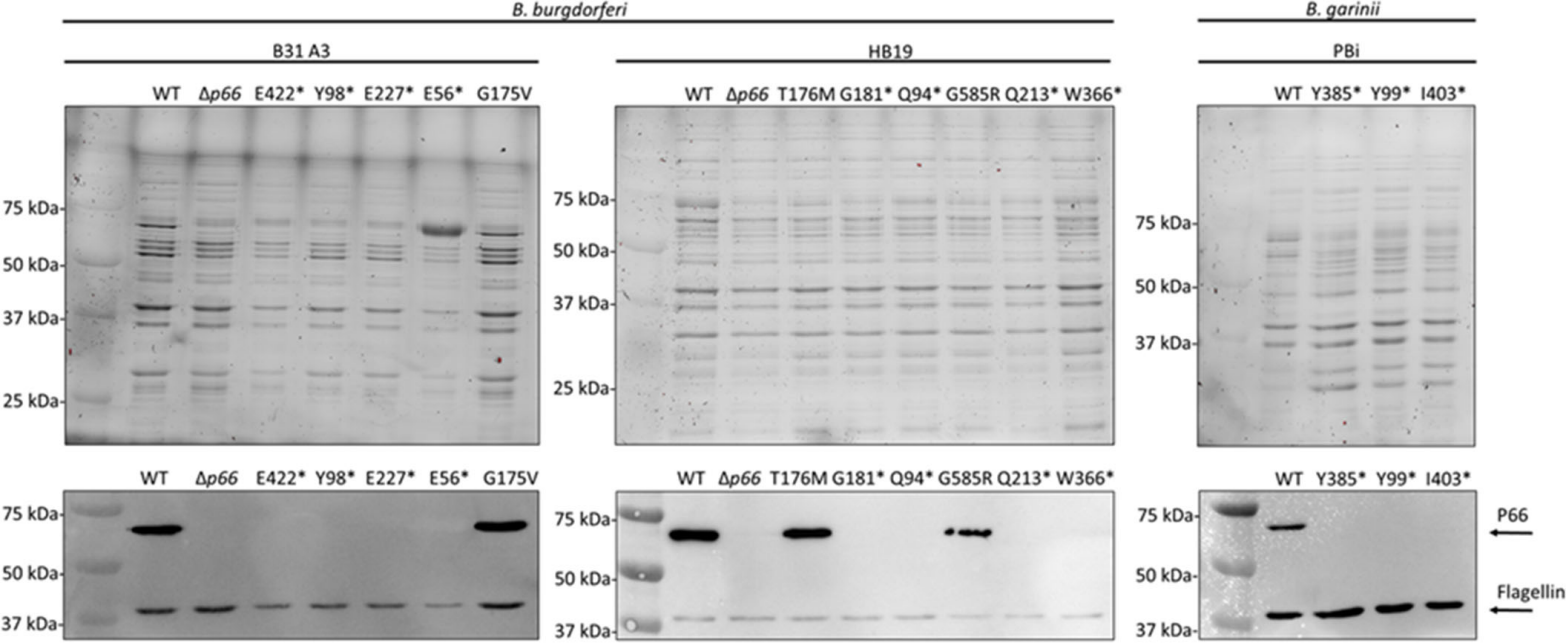
*In vitro* exposure of WT *B*. *burgdorferi* and *B*. *garinii* to vancomycin causes spontaneous *p66* mutations. *B*. *burgdorferi* B31 A3, *B*. *burgdorferi* HB19, and *B*. *garinii* PBi were grown to exponential phase, subcultured in the presence of vancomycin, and monitored for growth by darkfield microscopy. When exponential phase was again reached, the culture was plated in the presence of vancomycin for individual colonies. These colonies were cultured and the *p66* locus was sequenced. Representatives of all unique *p66* mutations obtained are shown (lane labels describe the P66 mutations; *denotes a change to a stop codon). Corresponding stain free images (top) and immunoblots (bottom) probed for P66 and flagellin are shown.

Given the P66-dependency of vancomycin sensitivity in *B. burgdorferi*, we hypothesized that vancomycin treatment could be used to generate novel *p66* mutants. WT and *Δp66* strains in the B31 A3 and HB19 backgrounds were cultured in the presence and absence of 1 μg/ml vancomycin for 2 weeks (**Figures 2A, B**). At the early time points, the WT strains demonstrated vancomycin sensitivity while the *Δp66* strain demonstrated resistance. However, growth was observed in the vancomycin-treated WT cultures at later time points. The same general trend was consistent for both *B. burgdorferi* backgrounds although the growth kinetics differed. To determine if the apparent developed resistance was due to altered P66 production, an immunoblot was performed on lysates from these cultures. Immunoblotting revealed that the vancomycin-treated WT cells had lost P66 production after two weeks (**Figures 2C, D**). Those cultures were no longer clonal so outgrowth was plated and individual colonies were propagated for further characterization. In the majority of isolates, immunoblotting revealed a loss of P66 production (**Figure 3**). Because P66 transcript and protein are unperturbed by *in vitro* temperature and pH conditions selected to mimic mammalian or tick environments (Cugini et al., 2003), we looked for genomic mutations in the *p66* gene. PCR-amplification and sequencing of the *p66* locus in each of the clones revealed P66 nonsense mutations (**Figure 3**). Sequencing revealed that some of the clones were identical siblings. This experiment was repeated several times to yield the spontaneous P66 mutants shown in **Figure 3** (i.e. E56*, Q94*, Y98*, G181*, Q213*, E227*, W366*, and E422*). Although most clones lost P66 production attributable to nonsense mutations, two unique clones, P66 T176M and P66 G584R, that maintained P66 production and harbored *p66* missense mutations, arose in the HB19 background (**Figure 3**). The P66 G175V isolate arose in the B31 A3 background following the high-throughput method described in this work. One representative of each unique isolate is shown (**Figure 3**). Note that numbering of P66 residues in this paper begins after the cleavable 21 aa signal sequence, which is representative of the biologically relevant mature protein (Bunikis et al., 1995; Bunikis et al., 1996). Nonsense mutations were not clustered in any particular region of *p66* but were identified throughout the coding sequence. Of note, none of the vancomycin-treated isolates maintained the WT *p66* allele.

**Figure 4:**
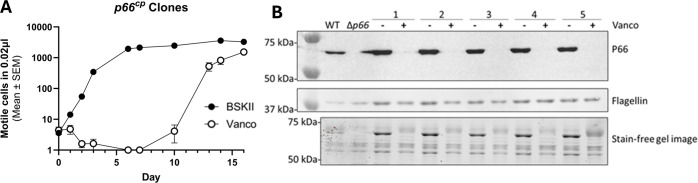
P66 complementation is sufficient to restore vancomycin sensitivity. B31 A3 vancomycin resistant clone P66 E442* was complemented on a plasmid (*p66^cp^*) in this study with an intact *p66* gene cloned in the shuttle vector pBSV2G, which encodes gentamicin resistance (Ristow et al., 2012). Five successful transformants were grown to exponential phase and subcultured in the absence of and presence of 1 μg/ml vancomycin for approximately 2 weeks. Culture density was monitored by darkfield microscopy **(A)**. Subsequently, cells were pelleted, washed, and protein extracts were separated by SDS-PAGE. Shown are the stain free image and immunoblot that was probed for P66 and flagellin production **(B)**.

We selected one of the spontaneous *p66* nonsense mutants (i.e. P66 E442*) and restored P66 production from a plasmid using a promoter which drives overproduction of P66 (Ristow et al., 2012). Five of these transformants were grown to exponential phase and subcultured with and without 1 μg/ml vancomycin. Sensitivity to vancomycin was restored at the early time points, but growth was observed in the vancomycin-treated cultures at the later time points (**Figure 4**). These strains had another loss of P66 production that was determined to be caused by mutations incurred on the plasmid-encoded *p66* gene. These data demonstrate that loss of WT P66 production is sufficient to permit growth of *B. burgdorferi* B31 A3 in the presence of vancomycin *in vitro*.

### 3.3 *p66* mutation following vancomycin treatment is conserved in another *Borrelia* species

P66 is conserved among all *Borrelia* species and known to be essential for mammalian infectivity of *B. burgdorferi* (Bunikis et al., 1995; Bárcena-Uribarri et al., 2010; Ristow et al., 2012; Curtis et al., 2022). To determine whether the spontaneous *p66* mutations under vancomycin pressure would similarly occur in another species, we also examined *B. garinii* PBi, a European Lyme disease-causing species. Growth of *B. garinii* (subsp. bavariensis) PBi *in vitro* is not as robust as the *B. burgdorferi* strains so extended incubation times and a reduced vancomycin concentration (0.5 µg/ml) were necessary to isolate vancomycin resistant mutants. The vancomycin-treated isolates exhibited a loss of P66 production and harbored nonsense mutations in *p66* (**Figure 3**). Taken together, spontaneous *p66* mutations arose in all tested *Borrelia* species and strains following prolonged vancomycin treatment.

### 3.4 High throughput method for generating vancomycin resistant clones

*P66* missense mutations had the potential to uncover novel information regarding porin function but were rarely identified. Therefore, we developed a high-throughput method for developing vancomycin resistance mutants. We attempted to use a 96-well plate format to simultaneously set up a large number of independent cultures. After two weeks, 23 wells from a single plate showed growth in the vancomycin-containing medium. Of the 23 isolates, only one maintained protein production and was identified to have a P66 G175V mutation (**Figure 3**). The other clones were not sequenced as they were likely nonsense mutations. This provides proof of concept for large scale identification of mutants but requires further optimization.

### 3.5 P66 missense mutations alter P66 production, oligomerization, and porin function

**Figure 5:**
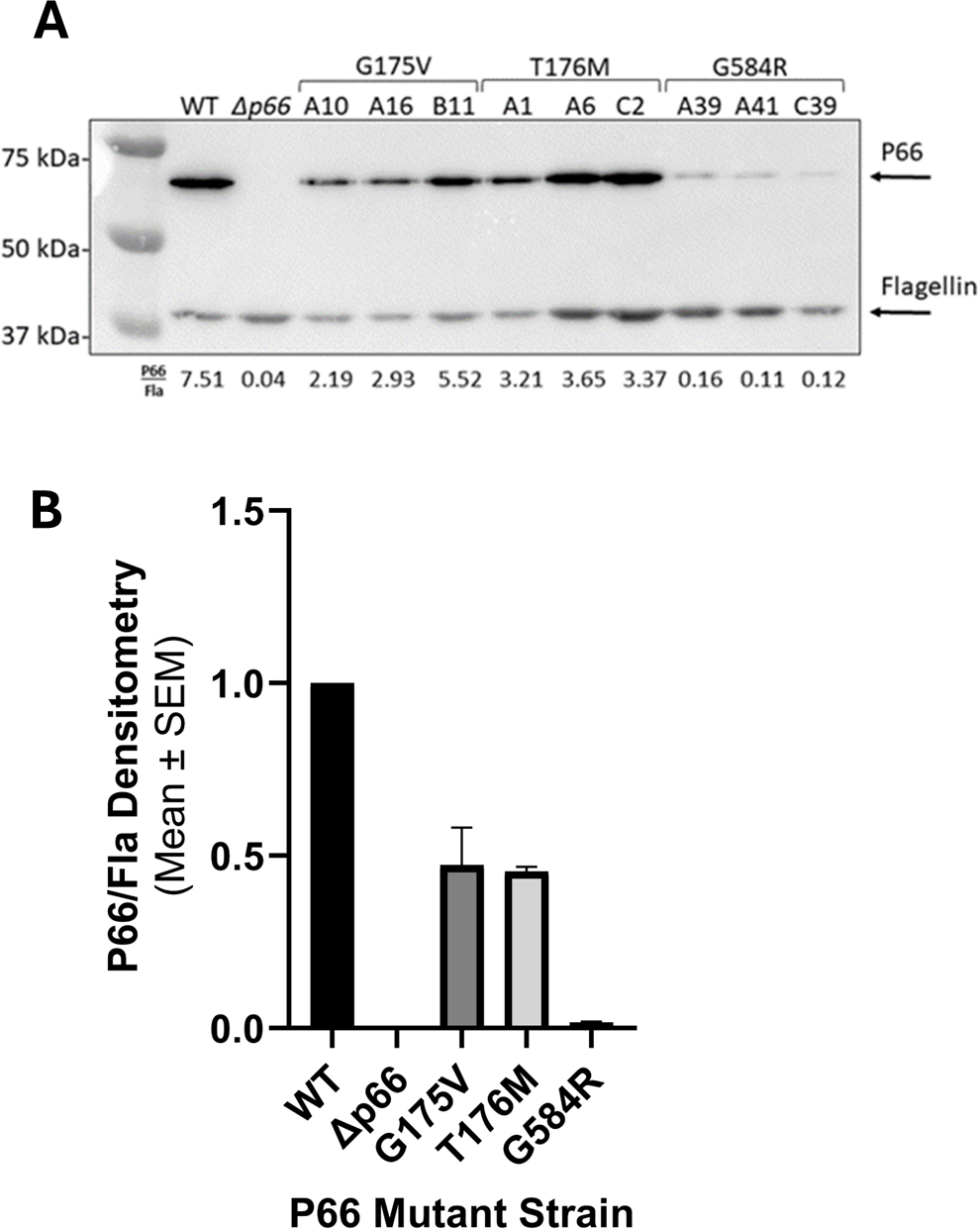
P66 production is reduced in the SDM missense mutants. Cell lysates of *B. burgdorferi* B31 A3 WT, *Δp66*, and three clones of each SDM missense mutant were subjected to SDS-PAGE and immunoblotting with antibodies against P66 and flagellin. In each lane, P66 was determined relative to its flagellin signal and densitometry shown below the blot **(A)**. The average for the triplicate clones was calculated and all values were normalized to WT **(B)**.

**Figure 6:**
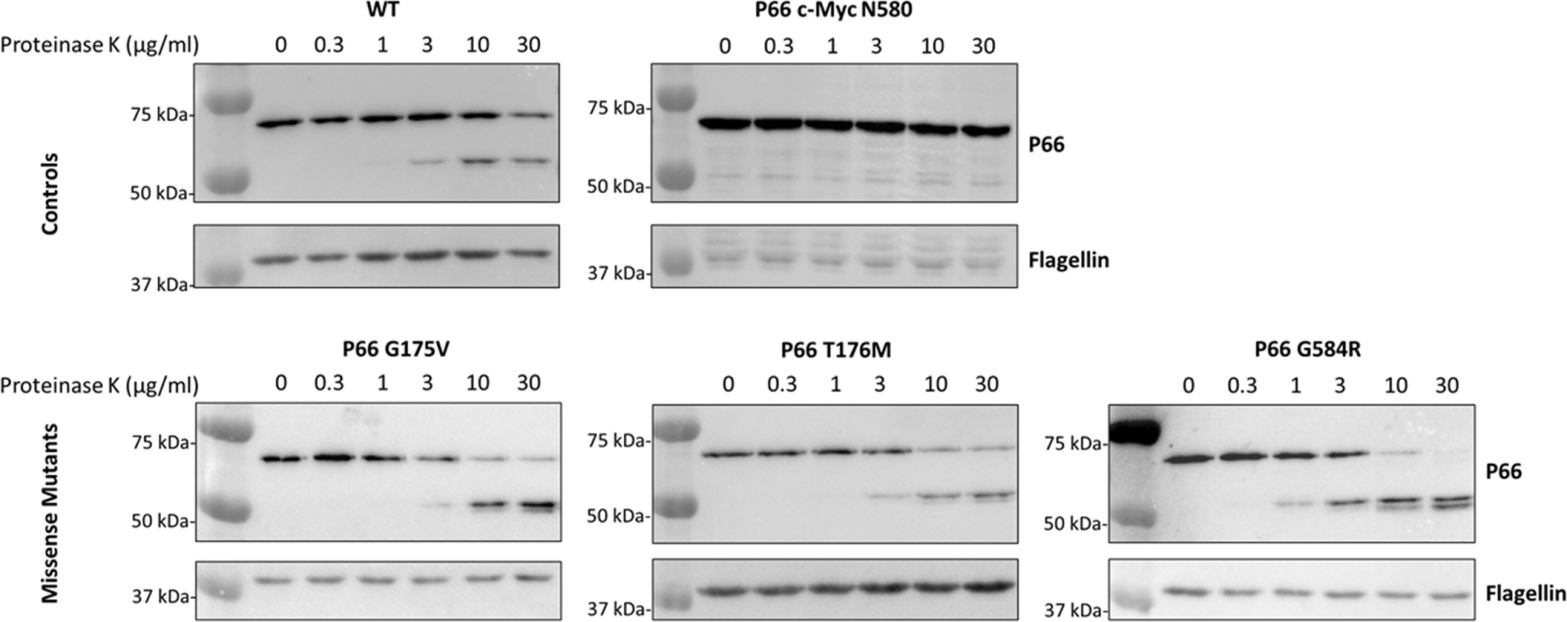
P66 is surface localized in the SDM mutants. Aliquots of *B*. *burgdorferi* B31 A3 WT, c-Myc N580^cp^, G175V, T176M, and G584R strains were treated with increasing concentrations of Proteinase (K) Proteinase K was inactivated by phenylmethanesulfonylfluoride and cell lysates underwent SDS-PAGE and immunoblotting for P66 and flagellin. Exposure times varied between antibodies and strains to optimize signals that would be conducive to densitometric analyses of each. In contrast to WT P66, P66 with a c-Myc tag insertion after N580 has previously been shown to be resistant to Proteinase K digestion (Curtis et al., 2022) and served as a control.

**Figure 7:**
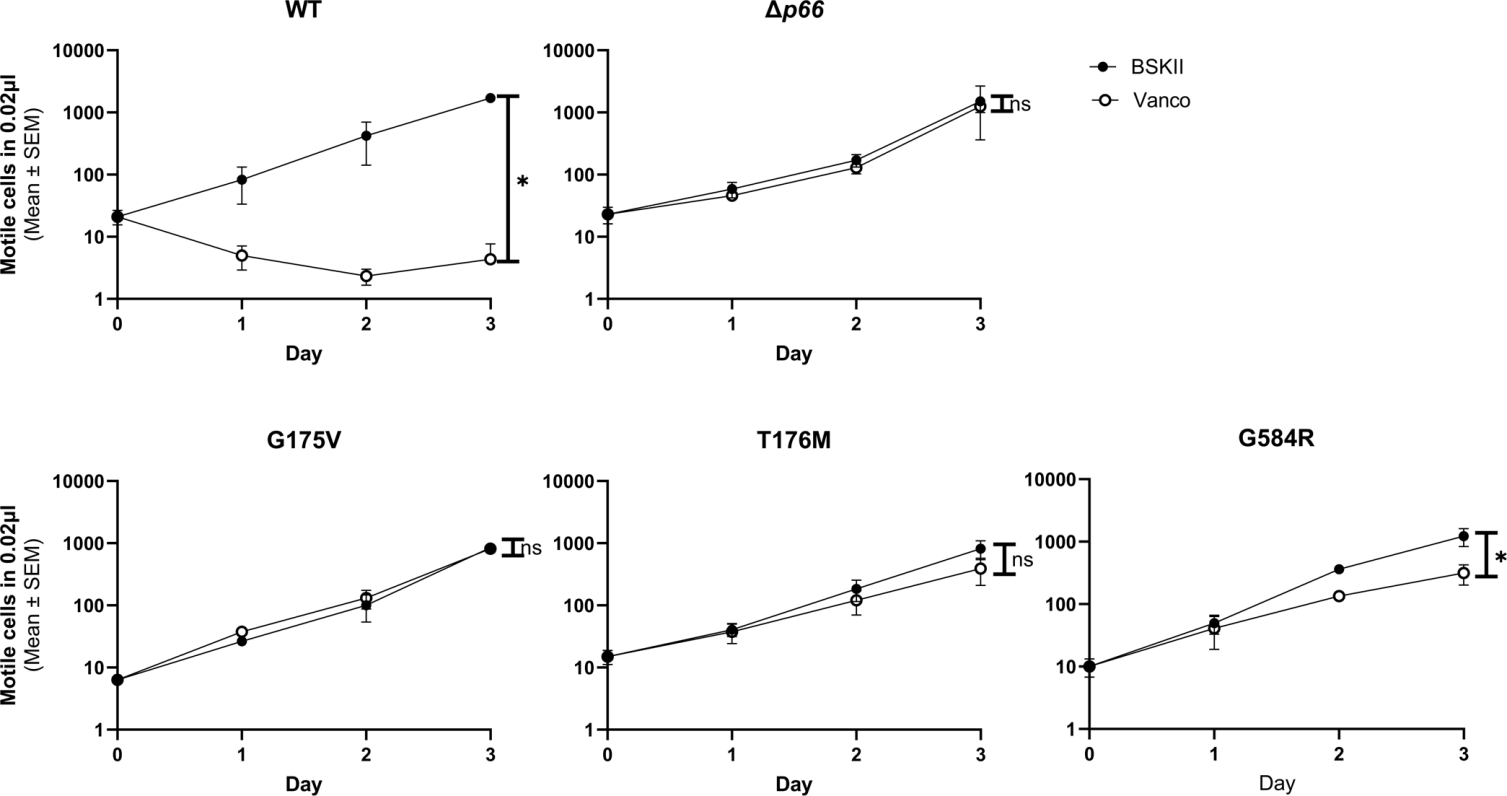
Missense mutants exhibit altered vancomycin susceptibility. Porin function was assessed by the vancomycin susceptibility assay for *B*. *burgdorferi* B31 A3 WT, *Δp66*, and three clones of each missense mutant. The mean and SEM of the three clones per missense mutant are shown. The mean and SEM of three independent trials is shown for WT and *Δp66* controls. GraphPad Prism 9.2.0 was used to analyze data by simple linear regression. The * denotes p <0.05 while ns (not significant) indicates p ≥0.05.

We next sought to determine whether P66 in the three isolates containing missense mutants (i.e. G175V, T176M, and G585R) was functionally different from WT. The isolates mimicked the *Δp66* strain in regard to minimum inhibitory concentration (MIC) and porin function as measured by vancomycin susceptibility as expected (**Supplementary Table 2**; **Supplementary Figure 1**). However, other mutations could exist in the genome. Therefore, site-directed mutagenesis (SDM) was used to generate these mutations in an infectious B31 A3 background to determine if the P66 mutations were solely responsible for these results. Note that residue G585 in the HB19 background aligns to G584 in the B31 A3 background. Immunoblotting revealed that P66 production was not equivalent in every mutant. Although G175V and T176M both produced P66 at ~50% wild type levels, G584R produced P66 at ~2% of the wild type (**Figure 5**). Surface localization of P66 in these mutants was verified by Proteinase K digestion (**Figure 6**). These SDM strains all mimicked the *Δp66* strain in terms of MIC and porin function as determined by vancomycin susceptibility (**Supplementary Table 2**; **Figure 7**).

**Figure 8:**
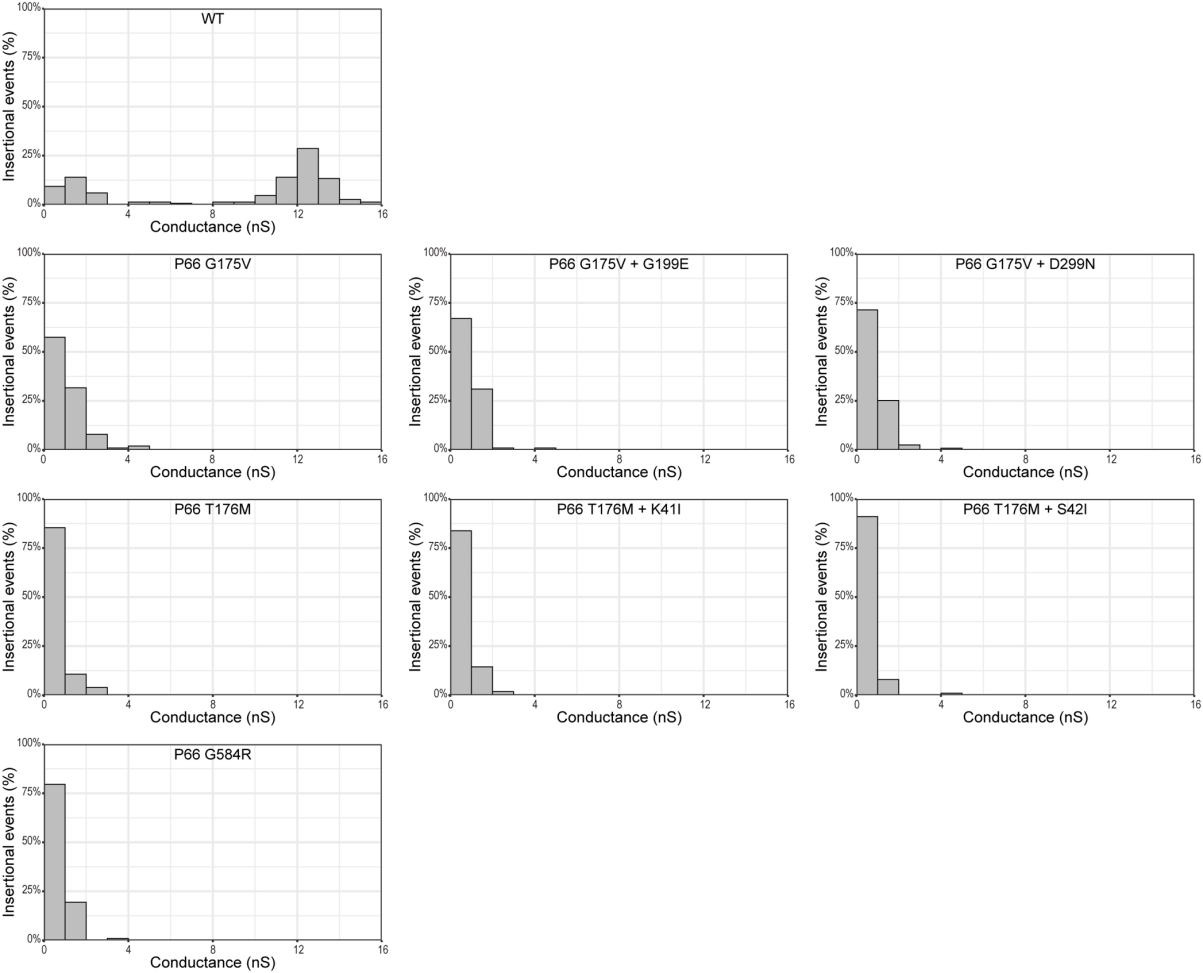
P66 porin function is altered for the missense and double mutants. B-fractions from the strains of interest were subjected to BLB. A minimum of 100 insertional events was obtained for each strain and the data are plotted as 1 nS bins.

**Figure 9:**
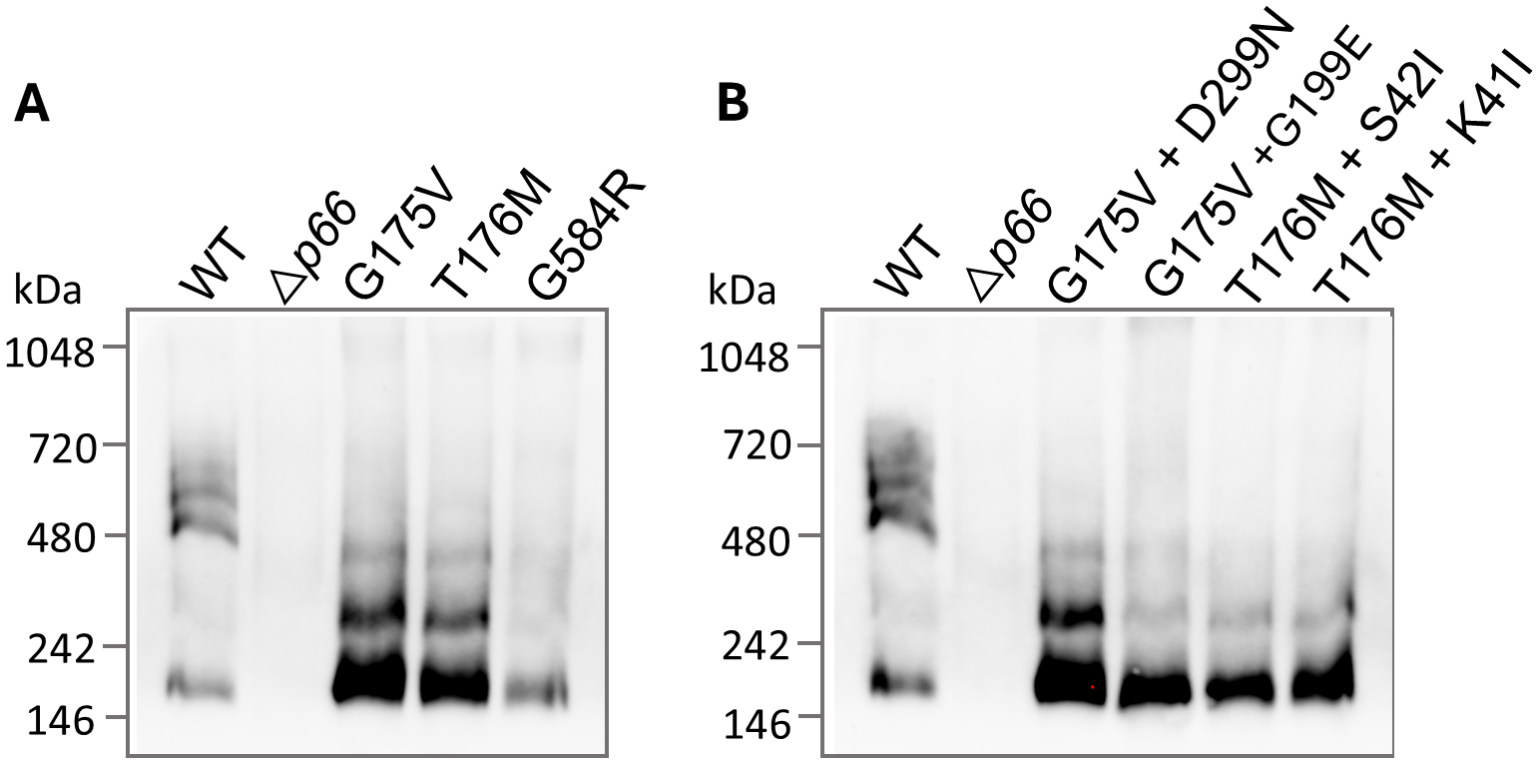
P66 oligomerization is disrupted in missense and double mutants. B-fractions from the strains of interest were subjected to BN-PAGE and immunoblotting for P66. WT, *Δp66*, the SDM missense mutants **(A)**, and the double mutants isolated from mouse tissues **(B)** are shown. All strains are in the B31 A3 background.

P66 porin function was also assessed in the SDM point mutants by the BLB assay, the gold standard method. In agreement with the porin function results found with the vancomycin screen, P66 porin function was observed to be disrupted with all insertional events less than 5 nS for the three missense mutants (**Figure 8**). This likely corresponds to the disruption of higher order oligomerization (e.g. heptamers) in favor of lower order oligomerization (e.g. trimers) as seen by BN-PAGE (**Figure 9A**). All three missense mutants exhibit disrupted oligomerization patterns with trimers representing the predominant oligomer and no heptamers visible. The P66 production in the G584R mutant is less than the other mutants, making visualization of higher order oligomers more difficult.

Integrin binding of the P66 missense mutants was attempted according to the method in described in Curtis et al., 2022. Unfortunately, no significant differences were observed, even between the positive and negative control strains. Therefore, we cannot conclude whether integrin-binding by P66 is affected by the missense mutations. Regardless, prior investigation into the integrin binding function of P66 determined that it is not essential for infectivity (Ristow et al., 2015). Therefore, it should not be a confounding factor in this study.

Taken together, these data demonstrate that the P66 missense mutations alter P66 production, oligomerization, and porin function but do not alter protein localization.

### 3.6 G175V and T176M mutants retain infectivity while G584R and nonsense mutants are attenuated

Given that the *Δp66* strain is not infectious, we hypothesized that the *p66* mutants isolated above would also be non-infectious. Two spontaneous nonsense mutants (E56* and E422*) were inoculated subcutaneously into groups of five mice at a dose of 1x10^5^ spirochetes per mouse. At 2 weeks post-infection, mice were euthanized, and tissues were harvested to inoculate liquid cultures. Cultures were monitored by darkfield microscopy for up to eight weeks for the presence of spirochetes. The results are shown in **Table 1**. As expected, no culturable spirochetes were recovered from either nonsense mutant or the negative controls, but all the mice infected with WT *B. burgdorferi* had recoverable spirochetes.

**Table 1:** Infectivity of P66 nonsense mutations and controls.

Inoculum	Blood	Bladder	Heart	Ankle	Skin	Ear	Brain	Total mice infected
Vehicle	0/5	0/5	0/5	0/5	0/5	0/5	0/5	0/5
WT	2/5	5/5	5/5	5/5	5/5	5/5	5/5	5/5
*Δp66*	0/5	0/5	0/5	0/5	0/5	0/5	0/5	0/5
P66 E56*	0/5	0/5	0/5	0/5	0/5	0/5	0/5	0/5
P66 E422*	0/5	0/5	0/5	0/5	0/5	0/5	0/5	0/5

Following characterization of the P66 missense mutants that were constructed by SDM, ID_50_ infections were performed. *p66^cc^*, which natively expresses the WT allele of P66 but also includes the gentamicin resistance cassette (Ristow et al., 2012), was used as an isogenic positive control to the SDM mutants. Mice inoculated with the vehicle control or *Δp66* were negative for recovery of spirochetes. Organ culture data is shown in **Supplementary Figure 2**. ID_50_ values for the other strains were calculated based on the number of infected and uninfected mice according to a Probit regression model (**Table 2**). The data for two independent clones of each missense mutant were combined. *p66^cc^* exhibited a higher ID_50_ than the WT control. The G175V and T176M mutants were not significantly different from the isogenic *p66^cc^* control, showing that these mutations did not negatively impact infectivity. In contrast, G584R was only recovered at 1x10^7^ and 1x10^9^ spirochetes/mouse from 6 total mice, showing a dramatic defect in infectivity which was statistically different from *p66^cc^*. As confirmation of these findings, a few isolates recovered from mice were screened for P66 production by immunoblot (**Supplementary Figure 3**) and sequence-verified to check for maintenance of the desired missense mutations. Importantly, all sequenced isolates had retained the targeted missense mutation through the course of infection (**Table 3**). Of interest, four of the sequence-verified isolates had gained additional missense mutations during infection (G175V + G199E; G175V + D299N; T176M + K41I; and T176M + S42I).

**Table 2:** ID_50_ values of P66 missense mutants.

Inoculum	ID_50_ (spirochete number)	Significance compared to WT	Significance compared to *p66^cc^*
WT	560	–	p <0.05
*p66^cc^*	6.48x10^4^	p <0.05	–
P66 G175V	5.18x10^5^	p <0.05	ns
P66 T176M	2.38x10^4^	p <0.05	ns
P66 G584R	6.53x10^8^	p <0.05	p <0.05

ID_50_ calculations were performed with a Probit regression model. Statistical significance was determined by T-tests and the raw p values corrected by the stepdown Bonferroni method. ns, not significant.

**Table 3:** Sequence verification of isolates recovered from ID_50_ experiment.

Mutant	Clone	Dose	Mouse #	Tissue	SDM retained	Additional mutation(s)
G175V	A10	10^7	2	Heart	Yes	None
	A10	10^7	4	Ankle	Yes	None
	A10	10^7	5	Ear	Yes	None
	A10	10^9	1	Ear	Yes	Asp299Asn
	A10	10^9	2	Bladder	Yes	Gly199Glu
	B11	10^6	1	Heart	Yes	None
	B11	10^6	1	Knee	Yes	None
	B11	10^7	1	Heart	Yes	None
	B11	10^7	5	Ear	Yes	None
	B11	10^9	3	Bladder	Yes	None
	B11	10^9	4	Ear	Yes	None
T176M	A1	10^9	1	Bladder	Yes	None
	A1	10^9	1	Ear	Yes	None
	A1	10^9	2	Ear	Yes	None
	A1	10^9	3	Ear	Yes	None
	A1	10^9	5	Bladder	Yes	None
	A6	10^9	1	Ear	Yes	Ser42Ile
	A6	10^9	3	Ear	Yes	None
	A6	10^9	3	Ankle	Yes	None
	A6	10^9	4	Ear	Yes	None
	A6	10^9	5	Ear	Yes	Lys41Ile
G584R	A41	10^9	5	Skin	Yes	None
	A41	10^9	5	Bladder	Yes	None
	C39	10^7	1	Ear	Yes	None
	C39	10^7	2	Ear	Yes	None
	C39	10^9	1	Ear	Yes	None
	C39	10^9	2	Skin	Yes	None
	C39	10^9	5	Skin	Yes	None

qPCR was also performed on infected mouse tissues to determine differences in bacterial burdens and dissemination of each mutant in the heart, tibiotarsus, and ear at the 1x10^7^ spirochetes/mouse dose which shows consistent infection for the positive control strains. No statistically significant bacterial burdens were detected between the point mutants and WT or *p66^cc^* strains (**Supplementary Figure 4**). However, the limited data for the G584R mutant limits statistical comparison for that mutant. The G175V and T176M P66 mutants were not attenuated in dissemination or bacterial burdens in a mouse model.

### 3.7 Spontaneous secondary point mutations were not compensatory for P66 porin function

**Figure 10:**
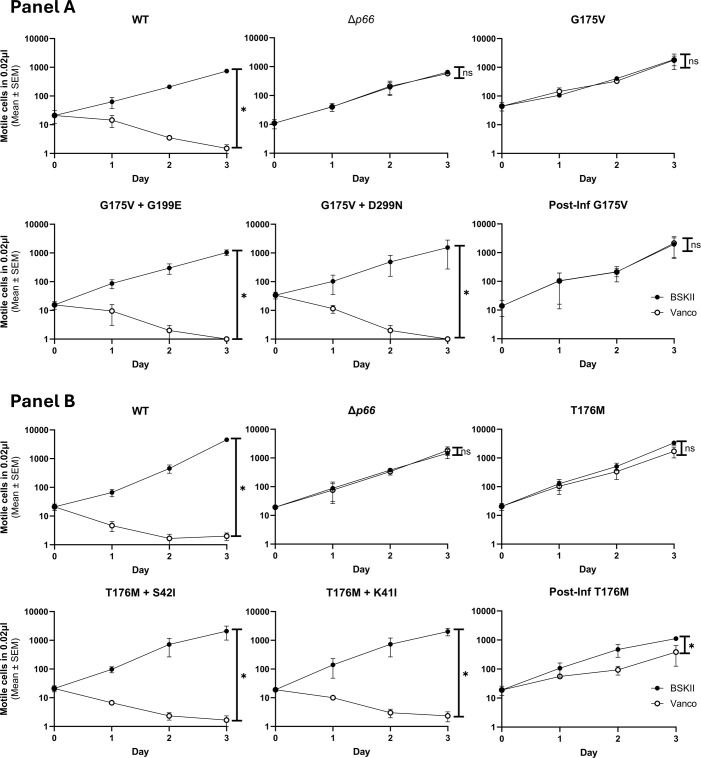
Double mutants mimic vancomycin susceptibility of WT. Porin function was assessed by the vancomycin susceptibility assay for B31 A3 WT, Δ*p66*, parental SDM missense mutant, and isolates recovered after mouse infections. The G175V strains are shown in Panel **(A)** and the T176M strains are shown in Panel **(B)** ‘Post Inf’ strains are post-infection isolates that did not gain *p66* mutations. This experiment was performed with ≥ three independent replicates; mean and SEM are shown. GraphPad Prism 9.2.0 was used to analyze data by simple linear regression. The * denotes p <0.05 while ns (not significant) indicates p ≥0.05.

Of interest, four of the sequence-verified isolates recovered from mice had gained additional missense mutations during infection. We evaluated P66 porin function in these strains. The double missense mutations more closely mimicked the WT than the parental strains and other post-infection isolates in terms of vancomycin susceptibility and MIC (**Figure 10**; **Supplementary Tables 2**, **3**). However, when BLB analyses were performed, the 12 nS signal characteristic of WT P66 was not restored (**Figure 8**). Furthermore, the secondary mutations did not restore the oligomerization profile to that of WT P66 (**Figure 9B**). In particular, P66 oligomerization for the G175V + G199E, T176M + S42I, and T176M + K41I strains appears more disrupted than their parental strains. This indicates that the additional mutations are not compensatory for P66 porin function and that the vancomycin screening method for porin function does not provide all the information required for full characterization of mutants.

### 3.8 P66 models are consistent with experimental results

**Figure 11:**
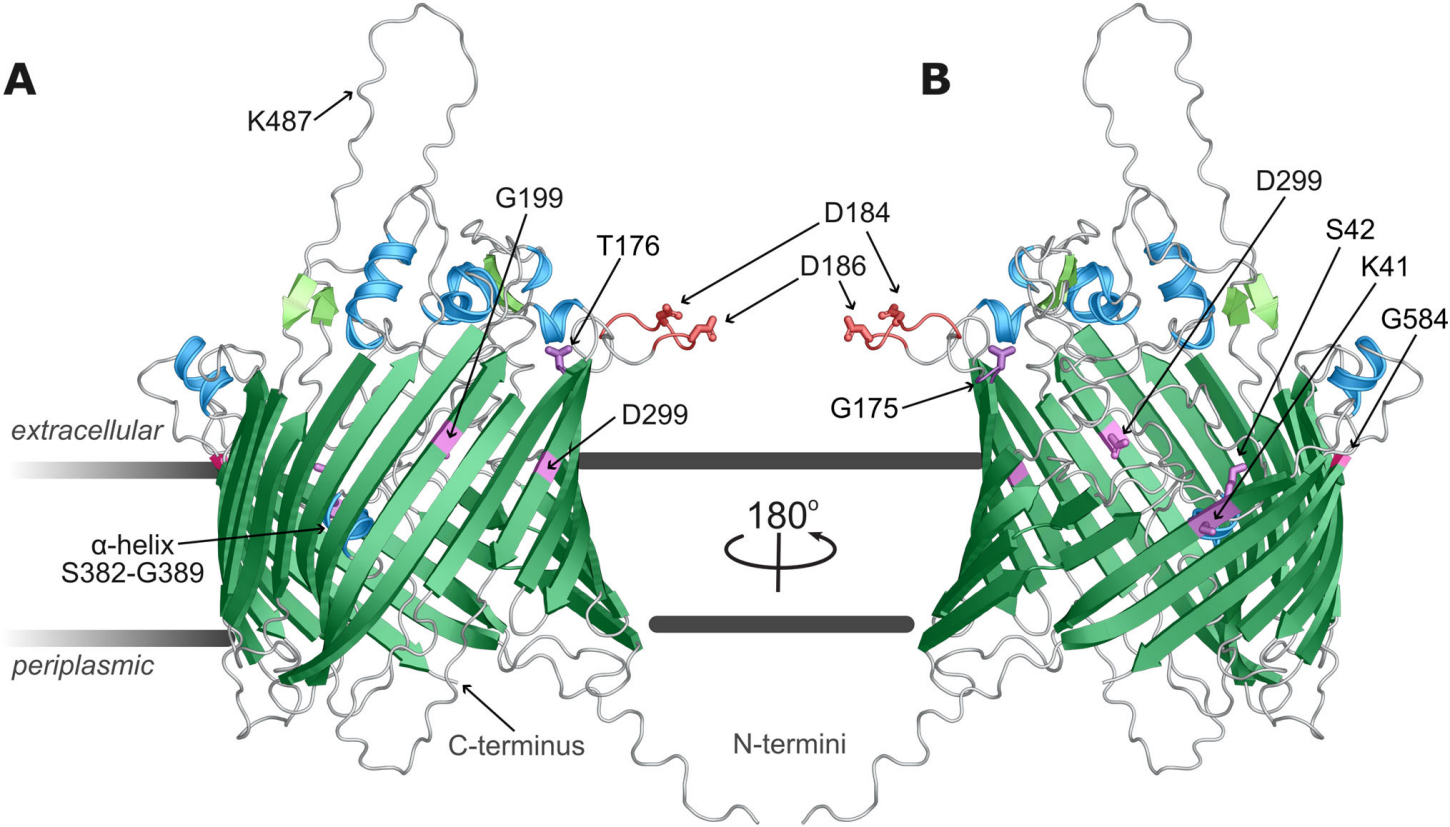
P66 domain prediction. The P66 *ab initio* structural model generated and ranked using DeepFold method and relaxed with AMBER force field is shown **(A, B)**. P66 adopts a transmembrane β-barrel conformation with ten extracellular loops, 22 antiparallel sheets with adjacent amino- and carboxy-termini. The model is colored by predicted secondary structure, with β-sheets in green, alpha helices in blue, loops in silver. In addition, residues K41, S42, G175, T176, G199, D299, and G584 are in violet, and the integrin binding domain is orange with D184 and D186 highlighted.

To better understand the potential impacts of the point mutations and secondary mutations in this work, the P66 native sequence was used to generate monomeric structure predictions (**Figure 11**) using AlphaFold2 with AMBER force field relaxation and enhanced neural network Deepfold algorithms (Jumper et al., 2021; Pearce et al., 2022). The 597 aa monomer was consistently modeled as a transmembrane beta-barrel (TMBB) porin containing 22 beta-sheets with adjacent N- and C-termini inserted towards the periplasmic space (**Supplementary Figure 5**). The consensus prediction is a model with a pore in the central area of the P66 monomer that opens obliquely opposite the largest extracellular loop (S462-F496), with multiple flexible domains overlying above and within the aperture. Predictions of the flexible loops are lower confidence by local distance difference test (lDDT) score (Mariani et al., 2013) than the transmembrane sheets, but indicate mostly unstructured domains, five extracellular alpha-helices, two pairs of beta-sheets (i.e. F321-K323/E334-F336 and Q460-S462/F495-D497), and a helix between S382-G389 collapsed within the TMBB monomer. The integrin-binding domain with exposed D184 and D186 [i.e. D205 and D207 in the unprocessed protein (Defoe and Coburn, 2001; Ristow et al., 2015; Kumar et al., 2015)] extends extracellularly as a flexible extracellular loop between T176 and Q194.

**Figure 12:**
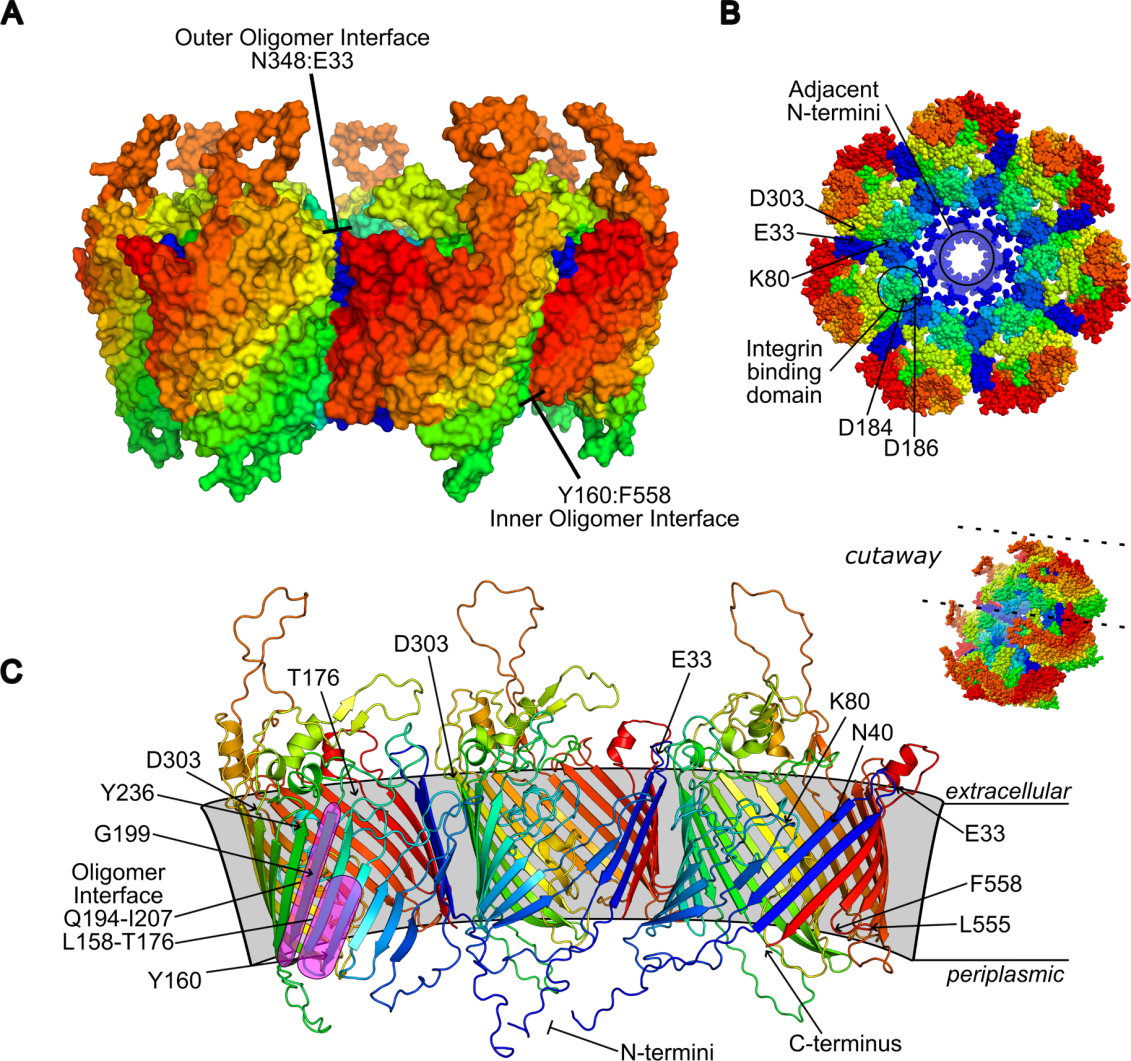
Proposed P66 oligomer. The highest ranked architecture by model quality scoring is heptameric with amino- and carboxy-termini centrally-oriented **(A, B)**. Shown is a focused depiction of three subunits of the heptamer, with proximal intermolecular residues indicated representing potential oligomeric interface **(C)**. Models are consistently colored blue to red from amino- to carboxy-termini.

Multimer predictions performed using GalaxyHomomer (Ko et al., 2012; Baek et al., 2017) algorithm resolved P66 folds in heptameric and octameric assembly. The highest ranked conformation by docking score and interface area was the heptamer in a conformation with proximal periplasmic N-termini (**Figure 12**). Three regions were suggested to contribute to oligomeric interaction by proximity of residues in the top-scored model: at the outermost leaflet involving the first and second extracellular loops including residues E33 and K80 with an opposing region bracketed by D303 and Y236; within the membrane between a broad interface comprised of the residues Q194-I207 and L158-T176 (notably containing G175 and T176), with Y198 having the closest side chain, and transmembrane sheet residues N40 and S42; and at the inner leaflet between the most C-terminal periplasmic loop L555-F558 and residues Y160, L165, and W204. The position of G584 is not predicted as proximal as residues like K80 and G175/G176 where direct intermolecular contacts would be made, but a bulky, basic substitution, in addition to synthesis and folding defects likely influences the adjacent L29 – P36 loop, disrupting oligomeric contacts around Y236. Similar contributions to structural integrity of the overall fold by G199, S42, and K41 are suggested by disrupted BLB signal. When mapped onto the model, G199 resides within the predicted transmembrane interface, and introduction of the carboxyl group of a glutamate substitution could cause direct hindrance, alter the Y198 side chain, or distort interactions at either leaflet. The Y198 residue is also significant to K41 and particularly S42, as in the heptamer model the hydroxyl groups are <3.0 Å apart. Distance measurements within the model show the centrally-oriented termini are collapsed to form a ring 22-24 Å in diameter, with individual subunit pore at least 7 Å, governed by inwards-facing side chains, particularly a trio of K41, K251, and K378.

## 4 Discussion

We hypothesized that the porin function of P66 was essential for the infectivity of *B. burgdorferi*. We concluded that *B. burgdorferi* exhibits P66-enhanced sensitivity and perhaps entry. However, we acknowledge that the MIC values for all strains remain low. This suggests that intact P66 porin function facilitates entry and sensitivity to vancomycin, but vancomycin can still enter the periplasm in the absence of P66 porin function at increased concentrations of drug, perhaps in a concentration-dependent manner. Evidence of the generalizability of this relationship between P66 and vancomycin is given by testing the HB19 *B. burgdorferi* background and *B. garinii* PBi in addition to B31 A3 *B. burgdorferi*. Because P66 is essential for infectivity and exhibits several domains that are conserved between Lyme disease- and relapsing fever-causing *Borrelia* species (Bárcena-Uribarri et al., 2010; Curtis et al., 2022), we anticipate that the relationship between P66 and vancomycin is widely generalizable. We further confirmed the P66 dependency of vancomycin sensitivity through a complementation experiment. A spontaneous vancomycin resistant *p66* nonsense mutant was transformed with a plasmid containing an intact *p66* gene. The complemented clones regained sensitivity to vancomycin. Furthermore, when the complemented clones were treated with vancomycin, they lost P66 production and contained mutations in the plasmid-encoded *p66* gene.

Prolonged culture of WT *B. burgdorferi* in vancomycin yielded mutants with an increased MIC to vancomycin. This is not surprising as antibiotic resistance of gram-negative, gram-positive, and acid-fast organisms can be generated in the laboratory (Gullberg et al., 2011; Martínez et al., 2011; Ismail et al., 2018). This principle has been known prior to the 1940s in the context of acquired spontaneous resistance to bacteriophages (Luria and Delbrück, 1943). Spontaneous mutations can be induced through serial passaging in increasing concentrations of antibiotic or through fluctuation assays. The latter method involves plating a clonal population on drug-containing medium and selecting for resistant clones (Luria and Delbrück, 1943). This method is dependent on the principle of continuous mutagenesis within an actively growing population of cells. The experiments described in this study are more similar to the fluctuation assay method for mutant generation than the serial passaging method. Upon isolation of putative *B. burgdorferi* mutants, we characterized them phenotypically for P66 production and genotypically for mutations in *p66*. Most mutants lost P66 production and harbored nonsense mutations in *p66*. However, a few isolates retained P66 production and harbored single missense mutations in *p66*.

While the nonsense mutants appear randomly dispersed throughout the protein sequence, it is interesting to note that some of the missense mutations identified in this study are in close proximity to each other in the primary protein sequence (e.g. G175 and T176, K41 and S42). Further experiments may shed light on whether these mutation locations are random or have significant bearing on interactions with vancomycin or novel functions of P66 *in vivo*.

It was not our goal to determine the frequency or rate of mutation in our experiments (Rosche and Foster, 2000), but the high throughput plate method is informative. Four microliters of exponential phase (≥5x10^7^ cells/ml) were inoculated into 200 µl of medium with vancomycin resulting in ≥200,000 cells per well. Not every well yielded mutants, but 23 of 94 wells did. Thus, nearly 25% of wells contained bacteria adapted through mutation. Mutations seemed to be selected for more consistently in the 5 ml culture format with multiple unique mutations arising within the same culture, perhaps because the starting population of cells is greater in this method.

While other publications explore persister cell formation and biofilms of *B. burgdorferi*, we do not consider the experiments of this paper to fall under those categories (Timmaraju et al., 2015; Feng et al., 2019; Hodzic et al., 2019; Rudenko et al., 2019; Sapi et al., 2019; Feng et al., 2020). Persister cells are defined by altered morphology and metabolic state (dormant rather than dividing) without corresponding genotypic changes (Lewis, 2007; Lewis, 2010; Rudenko et al., 2019). Furthermore, persister cells do not divide in the presence of antibiotic and, if antibiotic is removed, subsequent generations are again susceptible to the antibiotic (Balaban et al., 2019). In this work, the cells we observed grew actively (not aggregated) and retained spirochete morphology and motility, much like the observations of Harman et al. when *B. burgdorferi* was treated with ≤2 µg/ml vancomycin (Harman et al., 2017). Furthermore, we identified stable genotypic changes that accounted for the phenotypic changes we observed in P66 production and MIC alterations.

Vancomycin was used in our studies because we observed reproducible differences between WT and *Δp66* strains upon treatment *in vitro* that were useful for screening porin function. Other tested antibiotics, like ampicillin, showed no P66-dependency. We suspect that this is due to the sizes of these molecules, P66 being presumably ~0.8 nm at its narrowest (Barcena-Uribarri et al., 2013) and vancomycin being a rather large antibiotic (~1450 g/mol) in comparison to ampicillin (~350 g/mol). In our hands, vancomycin became a tool for screening P66 porin function and generating unique P66 mutants that could be used to address our hypothesis. Other groups have explored the idea of treating Lyme disease with vancomycin (Wu et al., 2018), but we do not advocate for this idea for reasons including serious side effects, need for placing an IV catheter and careful monitoring, and lack of data demonstrating better efficacy than other antibiotics (Wormser and Barbour, 2019; Lantos et al., 2021), and our results demonstrating that infectious vancomycin-resistant mutants may arise.

We demonstrated that the missense mutants (G175V, T176M, and G584R) were each sufficient to alter P66 porin function, decrease P66 production levels, and increase the MIC to vancomycin compared to the WT strain. Because they exhibited altered porin function, we tested the infectivity of the *B. burgdorferi* strains that produce P66 bearing these mutations in mice in ID_50_ experiments. The G175V and T176M mutants exhibited infectivity comparable to the *p66^cc^* strain despite producing P66 at approximately half the amount as the WT. These data do not support our hypothesis that the porin function of P66 is essential for *B. burgdorferi* infectivity. However, it is important to consider the native levels of P66 protein as underproduction likely decreases infectivity (a possibility for G584R) but overproduction of P66 from a plasmid copy has also been shown to nullify *B. burgdorferi*’s infectivity (Ristow et al., 2012). The regulation of *p66* expression is not entirely understood, although evidence supports the involvement of the Hbb transcription factor (Medrano et al., 2009). One strength of this study is the use of the native *p66* promoter for the mutant constructs during infection. The G584R mutant exhibited very poor infectivity, but P66 protein production in three clones was only ~2% of the WT (**Figure 5**). This may be a background strain difference as reduced production of P66 was not obvious in the spontaneous HB19 mutant, nor was the promoter mutated (**Figure 3**). This decreased production, rather than the amino acid change itself, may account for its infectivity defect. More experiments, possibly with a construct designed to overproduce this P66 G584R, would determine if this is the case. Notably, P66 with a c-Myc epitope tag inserted C-terminally to residue N580, only a few residues away, prevents surface localization of the protein (Curtis et al., 2022). It is possible that C-terminal β-barrel insertion sequences are affected leading to reduction of correctly inserted P66 in the outer membrane (Noppa et al., 2001; Paramasivam et al., 2012; Rigel and Silhavy, 2012; Kenedy et al., 2016). However, despite decreased levels of P66 production, the G175V and T176M mutants still exhibited infectivity comparable to the positive control.

The porin function of the P66 mutants was profiled by BLB. The BLB assay is a well-established biophysical technique to analyze and characterize pore-forming proteins in an artificial membrane. Still, it is an *in vitro* assay that does not fully correspond to the natural environment of the pores studied. In our case, the lipid composition in the artificial lipid membrane is not the natural *B. burgdorferi* membrane. In addition, other proteins that may be of importance for a pore’s natural environment are not taken into consideration. The environment overall is not that of the bacterial cell or the external milieu. Although these factors should be kept in mind when interpreting the BLB assay results, the BLB system provides a quantitative measure of porin function. The B-fractions used in this study contain additional *B. burgdorferi* porins that account for signals in the 0-4 nS range, clearly seen in the *Δp66* profile. This assay does not determine which signals may result from low order oligomers of P66 as opposed to other small porins. Interestingly, while both the BLB profiles and the vancomycin susceptibility assays indicated that P66 porin activity is altered in the G175V, T176M, and G584R mutants, contradictory results are observed for the double mutants. This may be a uniquely vancomycin-based anomaly as vancomycin was used both to apply mutational pressure as well as for screening the functional outcome. It is interesting that the second mutation appears to negate the first mutation but only for effects related to vancomycin and not for P66 oligomerization, porin function, or spirochete infectivity. However, the BLB results demonstrate that the secondary mutations do not truly restore full porin function. Although the screen for vancomycin resistance yielded *p66* mutants that were informative regarding porin function, which allowed us to test the role of porin activity in the mouse model of infection, vancomycin resistance is not necessarily reflective of P66 porin function. This is also important to note as a limitation to the vancomycin susceptibility screen; confirmation through BLB remains critical to quantitatively assess P66 porin function.

P66 oligomerization is associated with porin function. As seen in Curtis et al., 2022, it is likely that higher order P66 oligomers (e.g. heptamers) exhibit high channel conductance insertional events in the BLB while lower order oligomers and monomers exhibit low channel conductance insertional events (Curtis et al., 2022). Consequently, changes in P66 porin function are expected to be reflected in altered oligomerization profiles, which may also reflect destabilization of the complex. If possible, it would be interesting to generate mutants that maintain higher order oligomerization while porin function is disrupted in order to tease apart the contributions of these variables to infectivity. From the predicted positioning of residues in the model, mutations at K251 or K378 may have less effect on oligomerization as they are incorporated into flexible loops rather than the transmembrane beta sheets. The oligomer may be able to affect host responses to *B. burgdorferi* attachment to, for example, integrins on the cell surface by promoting the clustering of host cell receptors. Again, this possibility can be pursued in future studies.

We also present updated predicted monomer and multimer models of P66. Previous sequence-based, *in silico* computational analyses of P66 have included membrane topology with PRED-TMBB and secondary structure predictions with TMBpro, describing P66 as an amphiphilic, pore-forming TMBB outer membrane protein (Kenedy et al., 2014; Curtis et al., 2022). The modeling strategy employed here incorporates advancements in computational protein folding that corroborate these initial predictions and add significant resolution to a challenging target with little homology to other solved structures (Dunn et al., 2015). In addition to the static structures, intramolecular motions are anticipated, especially of the flexible loop domains, to regulate dynamic binding and porin functions of P66 and alter the structure of the individual P66 subunits and the resulting geometry of the higher order complexes.

Among multimer predictions, heptamer and octamer structures were highest ranked by docking score. Lower or higher-order oligomers did not maintain a consistent fold but were also missing the contribution of the outer membrane lipids. The lower-tier heptameric conformation, with N-termini facing outwards, had a low-confidence central opening of ~70 Å and steric clashes among extracellular units. The most energetically favorable model among multimeric assemblies is the heptamer with N-termini centrally oriented. Modeled proximity is an incomplete indicator of regions that form oligomeric contacts, but the overall prediction suggests the monomers are stabilized in a ring at multiple discrete sites. At the periplasmic leaflet, an inner interface forms between the final inwards-facing loop residues, with F558 closest to the Y160 residue of the adjacent unit. Mutagenesis of G199 may affect further contacts within the membrane, as it likely perturbs the function of Y198, a central residue approached by residues N40, S42, and potentially K80 of the next monomer. At the extracellular leaflet, E33 of the first extracellular loop and K80 of the second may be in domains with enough flexibility to bridge to another monomer, potentially regulated by side chains of D303 and Y236. When c-Myc tags were inserted C-terminally to E33 and D303, both P66 mutants exhibited disrupted oligomerization with a predominance of trimers, disrupted porin function ≤4 nS, and intermediate vancomycin susceptibility profiles (Curtis et al., 2022). P66-dependent vancomycin sensitivity raises the possibility of direct interaction at the outer membrane. Vancomycin has a large molecular surface area and frequently oligomerizes in different supra-complexes (Jia et al., 2013), which could hypothetically interact with individual P66 porins or at a combined surface formed by the P66 multimer. The accumulating site-specific P66 functional and biochemical experimental data are reinforced by consistency with computational predictions of fold, conformation, and orientation of key amino acid residues. Progress towards targeting P66 to counter *Borrelia* pathogenicity will be enhanced by solving high-resolution three-dimensional structures of the purified P66 porin.

In conclusion, bacterial burdens of the P66 porin function mutants G175V and T176M do not differ from the WT in a murine model. These data provide evidence that *B. burgdorferi* can tolerate alterations to P66 porin function *in vivo*. The degree of tolerance to alterations of porin function could be further investigated as the BLB profiles of the mutants in this study were similar. Furthermore, future studies may investigate the biological substrates of P66. Given the size of P66, it is probable that relatively large substrates, such as peptides, gain access to the cytoplasmic membrane oligopeptide transporters through this porin (Bono et al., 1998; Kornacki and Oliver, 1998). Another possible function is the elimination of metabolic waste products. Finally, solving the structure of P66 will also shed light on its enigmatic but essential function in *B. burgdorferi* infection and ultimately Lyme disease pathogenesis. This knowledge could be a critical step toward improving therapeutics and prevention of Lyme disease.

## Data Availability

The original contributions presented in the study are included in the article/**Supplementary Material.** Further inquiries can be directed to the corresponding author.
