# Supplementary material for: Borrelia burgdorferi tolerates alteration to P66 porin function in a murine infectivity model
Source: Front Cell Infect Microbiol. 2025 Jan 21;14:1528456. doi: 10.3389/fcimb.2024.1528456 (PMC11790652; doi:10.3389/fcimb.2024.1528456)
Supplement: Supplementary file 1 [file DataSheet1.pdf]

Supplementary Material

1 Supplementary Figures

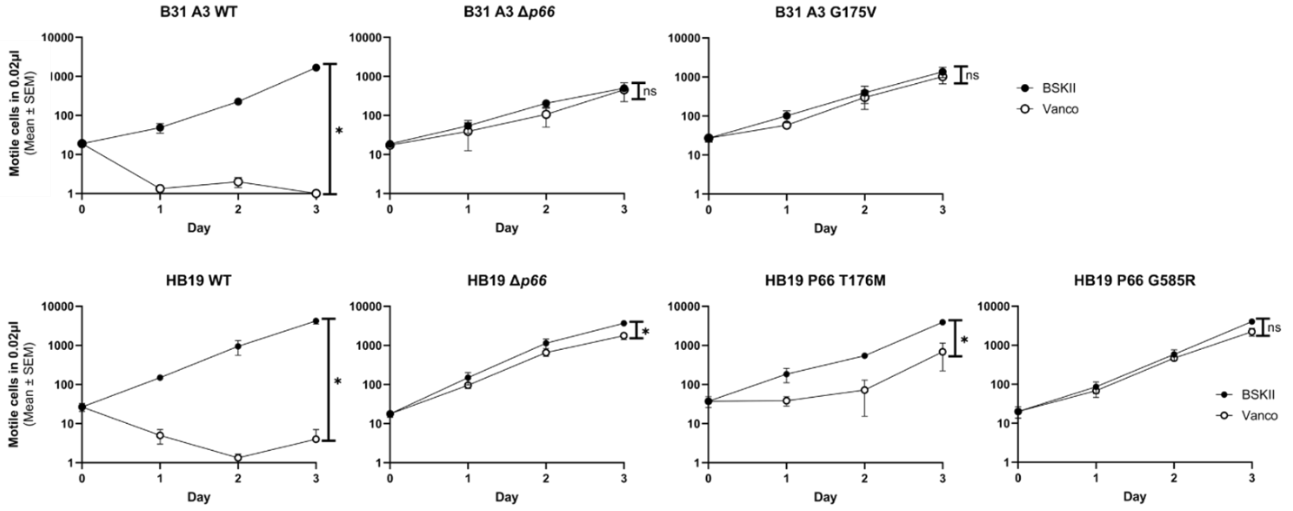

Supplementary Figure 1. Spontaneous P66 missense mutants from the B31 A3 and HB19 backgrounds exhibit altered P66 porin function. Porin function was assessed by the vancomycin susceptibility assay. Control strains (WT and Δ*p66*) and spontaneous P66 missense strains were grown to exponential phase in BSKII and diluted to $1x10^6$ cells/ml in the presence and absence of vancomycin. Culture density of motile *B. burgdorferi* was determined daily by darkfield microscopy on a Petroff-Hausser counting chamber for 3 days. The strains in the B31 A3 background are shown on the first row; each HB19 strain is shown on the second row. This experiment was performed with ≥ three independent replicates; mean and SEM are shown. GraphPad Prism 9.2.0 was used to analyze data by simple linear regression. * = $p < 0.05$.

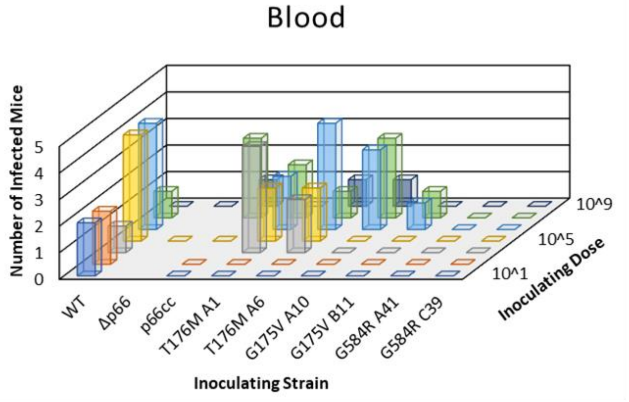
Blood
Number of Infected Mice
0
1
2
3
4
5
WT
Δp66
p66cc
T176M A1
T176M A6
G175V A10
G175V B11
G584R A41
G584R C39
Inoculating Strain
10^1
10^5
10^9
Inoculating Dose

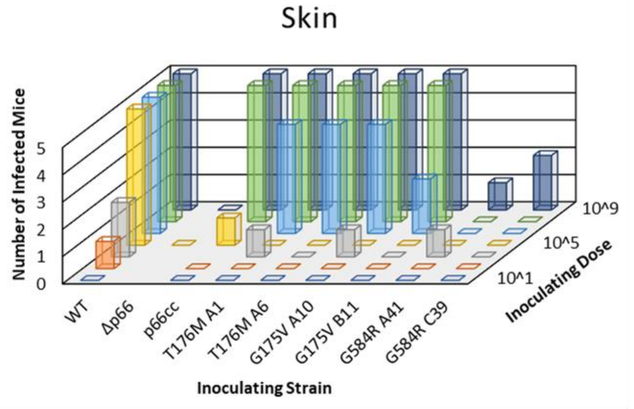
Skin
Number of Infected Mice
0
1
2
3
4
5
WT
Δp66
p66cc
T176M A1
T176M A6
G175V A10
G175V B11
G584R A41
G584R C39
Inoculating Strain
10^1
10^5
10^9
Inoculating Dose

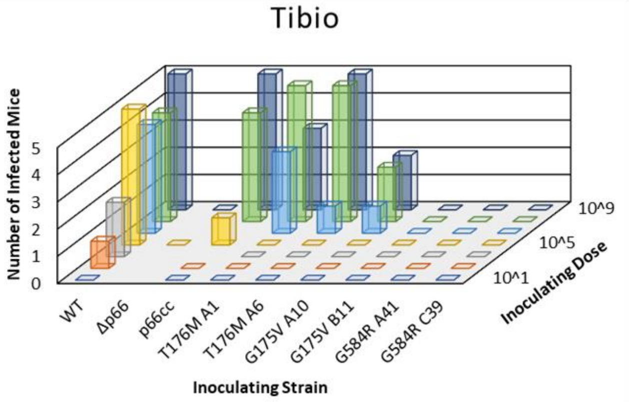
Tibio
Number of Infected Mice
0
1
2
3
4
5
WT
Δp66
p66cc
T176M A1
T176M A6
G175V A10
G175V B11
G584R A41
G584R C39
Inoculating Strain
10^1
10^5
10^9
Inoculating Dose

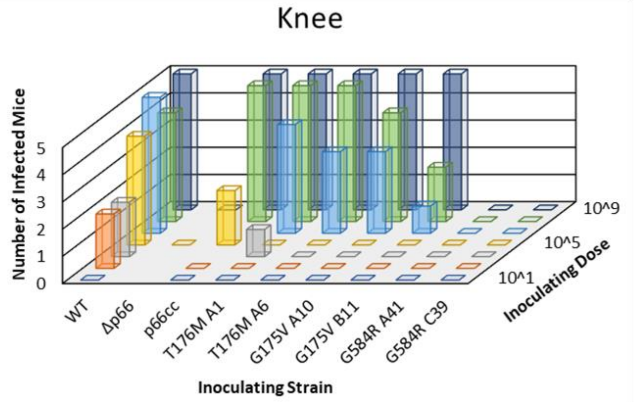
Knee
Number of Infected Mice
0
1
2
3
4
5
WT
Δp66
p66cc
T176M A1
T176M A6
G175V A10
G175V B11
G584R A41
G584R C39
Inoculating Strain
10^1
10^5
10^9
Inoculating Dose

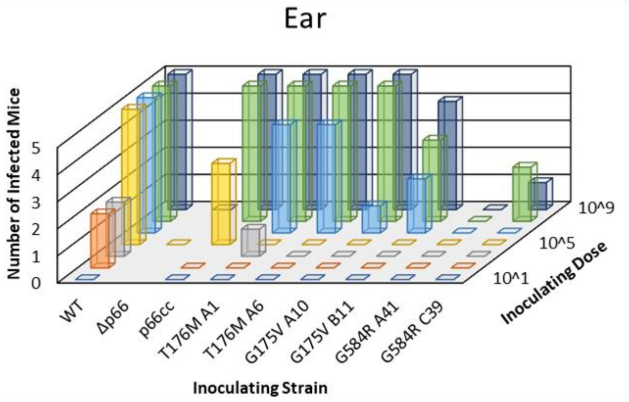
Ear
Number of Infected Mice
0
1
2
3
4
5
WT
Δp66
p66cc
T176M A1
T176M A6
G175V A10
G175V B11
G584R A41
G584R C39
Inoculating Strain
10^1
10^5
10^9
Inoculating Dose

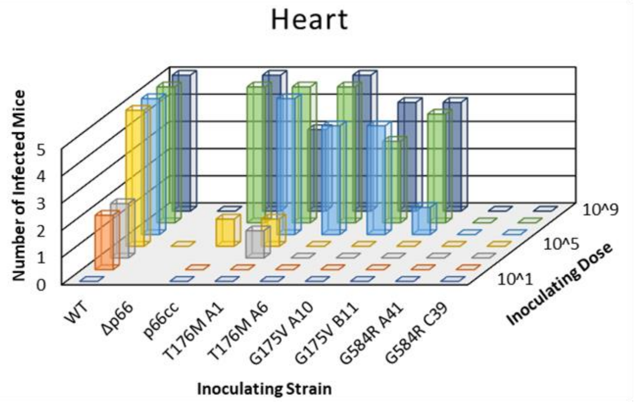
Heart
Number of Infected Mice
0
1
2
3
4
5
WT
Δp66
p66cc
T176M A1
T176M A6
G175V A10
G175V B11
G584R A41
G584R C39
Inoculating Strain
10^1
10^5
10^9
Inoculating Dose

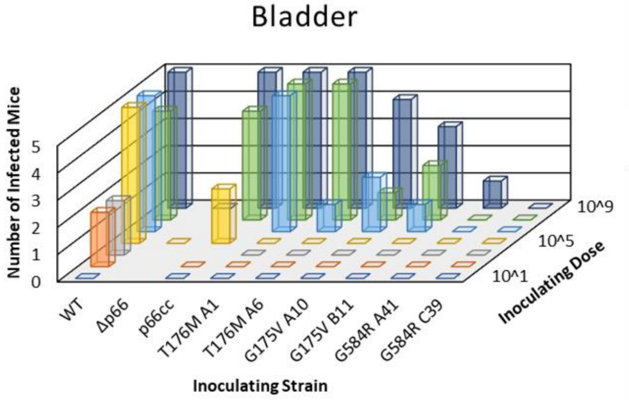
Bladder
Number of Infected Mice
0
1
2
3
4
5
WT
Δp66
p66cc
T176M A1
T176M A6
G175V A10
G175V B11
G584R A41
G584R C39
Inoculating Strain
10^1
10^5
10^9
Inoculating Dose

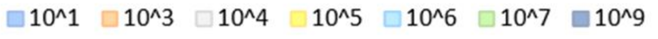
10^1
10^3
10^4
10^5
10^6
10^7
10^9

Supplementary Figure 2. The T176M and G175V mutants establish a disseminated infection in a mouse model while the G584R mutant does not. Five week old C3H/HeN mice were inoculated with a range of doses (10^1 to 10^9) of *B. burgdorferi* spirochetes harboring *p66* alleles of interest (two clones per P66 SDM mutant). At 4 weeks post-inoculation, mice were euthanized by CO_2 inhalation and the following tissues were collected: blood, inoculation site skin, ear, heart, bladder, tibiotarsus, and knee joint. The tissues were used to inoculate BSKII medium and these cultures were monitored for spirochete growth for up to 8 weeks. Culture positivity results are shown. The inoculating strains and *p66* mutations are shown along the horizontal axis. The vertical axis shows how many mice per group of five were culture-positive. The spirochete dose ascends from the front to the back of the graph by powers of ten with a unique color for each dose. Each graph shows the data for a single tissue type.

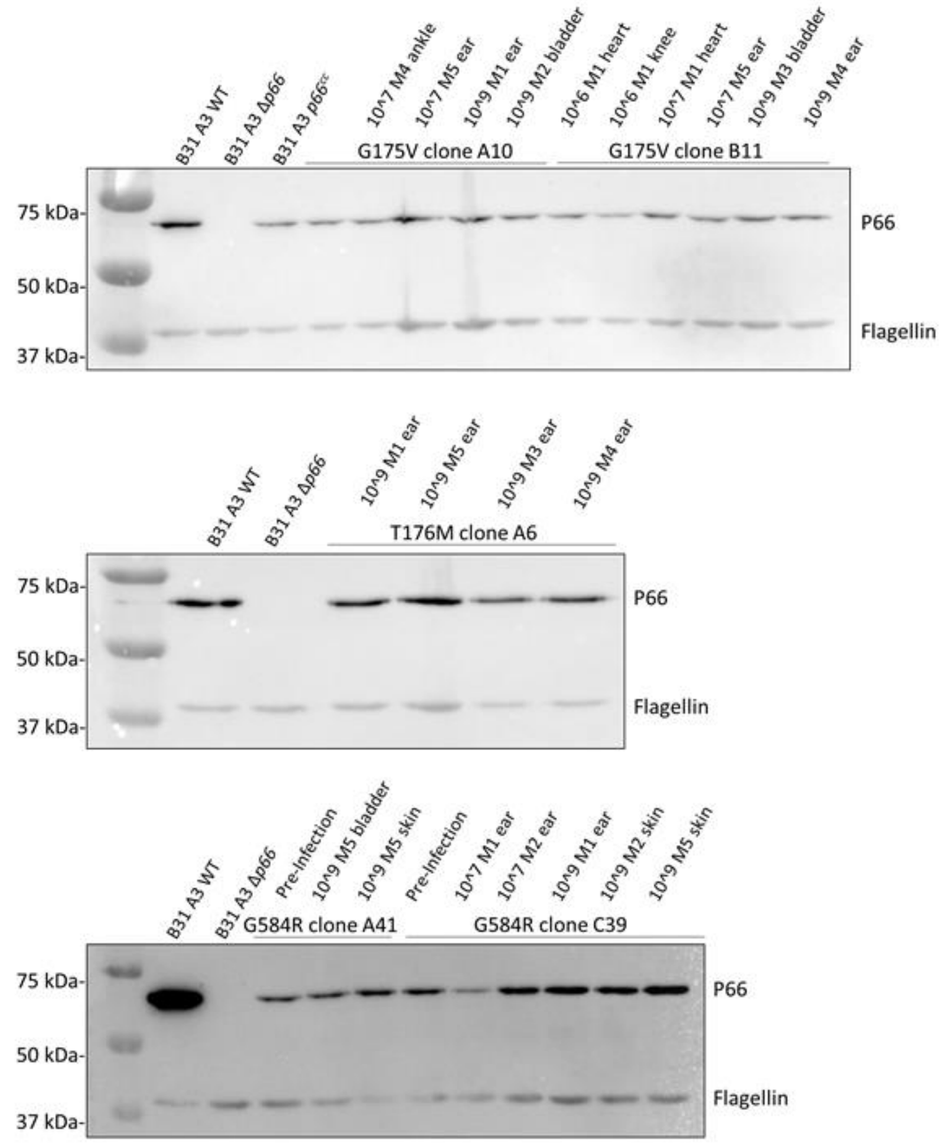

Supplementary Figure 3. Post-infection isolates produce P66 protein. At the conclusion of the ID_{50} experiment, some of the isolates recovered from mice were cultured for characterization (Table 3). Bacterial cell lysates were subjected to SDS-PAGE and western blotting for P66 and flagellin. Shown are P66 G175V (*top*), T176M (*middle*), and G584R (*bottom*) isolates. Labels indicate the dose of spirochetes inoculated into the mouse, the mouse identifier (M1 through M5), and the tissue from which the isolate was recovered. 'G175V clone A10 10^9 M1 ear' harbors a novel Asp299Asn mutation, 'G175V clone A10 10^9 M2 bladder' harbors a novel Gly199Glu mutation, 'T176M clone A6 10^9 M1 ear' harbors a novel Ser42Ile mutation, and 'T176M clone A6 10^9 M5 ear' harbors a novel Lys41Ile mutation. All other isolates retained the parental *p66* mutation only.

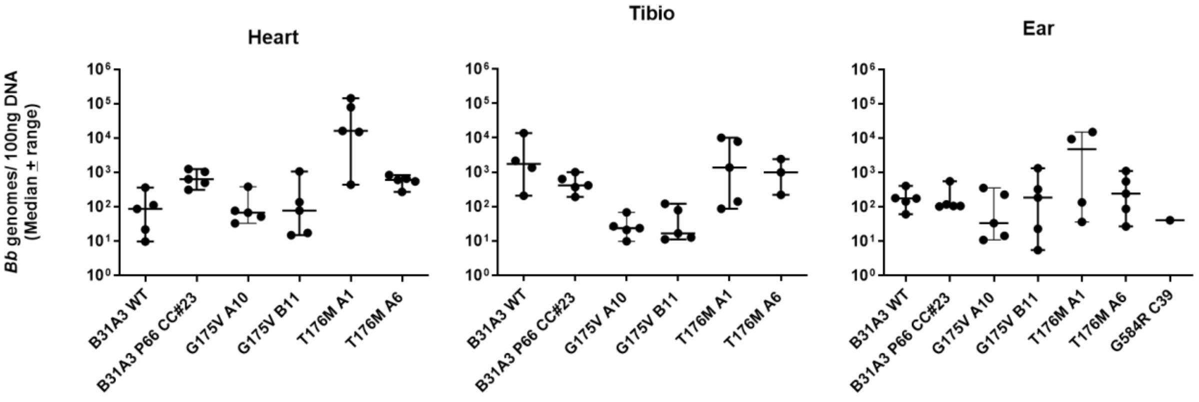

Supplementary Figure 4. Bacterial burdens of the G175V and T176M mutants do not differ from the WT in selected tissues. qPCR of infected mouse tissues was performed as previously described (Ristow et al., 2015). C3H/HeN mice were subcutaneously inoculated with a range of doses ($1x10^1$ to $1x10^9$ spirochetes/mouse) of WT, *p66^{cc}* #23, P66 T176M clones A1 and A6, P66 G175V clones A10 and B11, or G584R clones A41 and C39. Tissues were harvested 4 weeks post-inoculation and frozen. Total DNA was purified and analyzed by qPCR with primers to detect *Borrelia* genomes and mouse genomes. Bacterial burdens from mice inoculated with 10^7 bacteria are shown for all strains. The number of *Borrelia* genomes detected per 100 ng of extracted DNA are shown for each mouse (groups of 5). Groups with less than 5 points are due to culture-negative tissues at the given inoculum. Median and range are shown. Statistical analyses were performed using a one-way ANOVA Kruskal-Wallis test with multiple comparisons to the B31 A3 *p66^{cc}* #23 as the control column as well as a Benjamini, Kreiger and Yekutieli for false discovery. No p values of significance (i.e. $p < 0.5$) were detected.

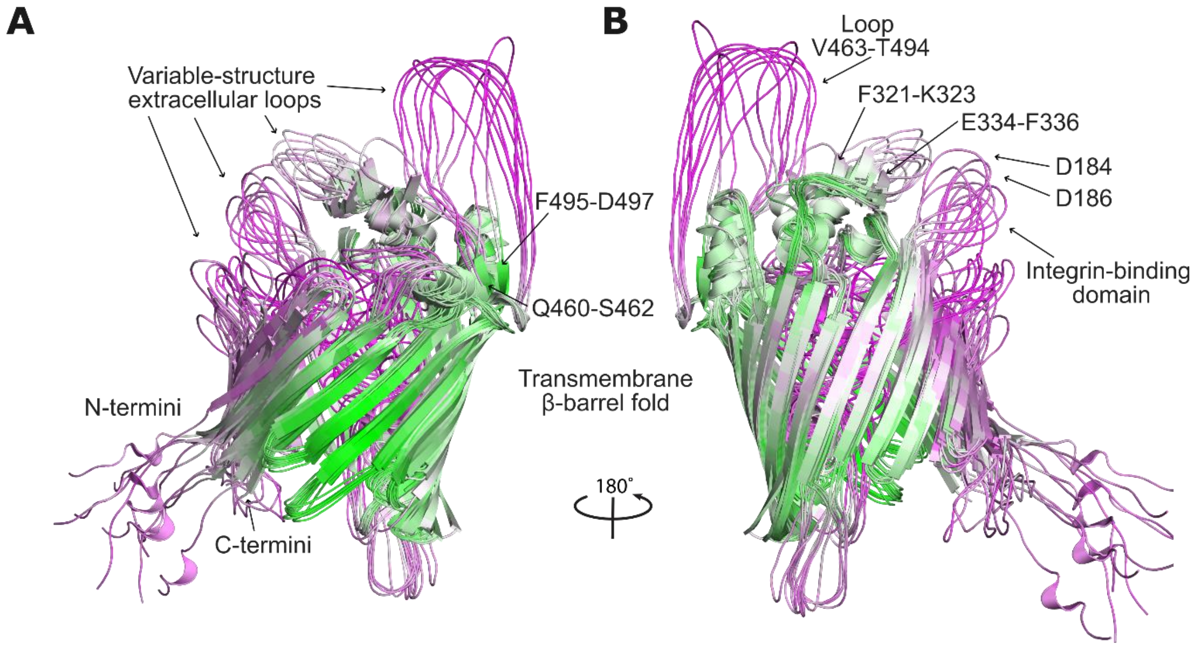

Supplementary Figure 5. P66 structure modeled with AlphaFold2. Overlays of iterative structure calculations are shown from different angles (A, B). Predictions define a β-barrel core domain with multiple unstructured extracellular loop domains and intracellular termini. The models are colored by lDDT score, showing increasing local model quality from magenta to green.

2 Supplementary Tables

2.1 Supplementary Table 1. Primers used in this study

Name	Sequence 5' -> 3'	Purpose	Reference
P66 G175V For	GAT TTC TGT TAC CCG TGA CTG TTC CCC CTA TTG CA	SDM	This study
P66 G175V Rev	TGC AAT AGG GGG AAC AGT CAC GGG TAA CAG AAA TC	SDM	This study
P66 T176M For	GAT TTC TGT TAC CCA TGC CTG TTC CCC CTA TTG C	SDM	This study
P66 T176M Rev	GCA ATA GGG GGA ACA GGC ATG GGT AAC AGA AAT C	SDM	This study
P66 G584R For	CAG AAT TGC AAG AAA GCA CTT CTA ATA GCA GCA TTA TTT GTG TTT T	SDM	This study
P66 G584R Rev	AAA ACA CAA ATA ATG CTG CTA TTA GAA GTG CTT TCT TGC AAT TCT G	SDM	This study
Ms β-Actin FWD	TCA CCC ACA CTG TGC CCA TCT ACG A	qPCR for infected tissues	(Ristow et al., 2015)
Ms β-Actin REV	GGA TGC CAC AGG ATT CCA TAC CCA	qPCR for infected tissues	(Ristow et al., 2015)
Rec A nTM17.F	GTG GAT CTA TTG TAT TAG ATG AGG CTC TCG	qPCR for infected tissues and integrin-binding assay	(Morrison Tom et al., 1999)
Rec A nTM17.R	GCC AAA GTT CTG CAA CAT TAA CAC CTA AAG	qPCR for infected tissues and integrin-binding assay	(Morrison Tom et al., 1999)
OLCR03	AAC TTG AAT CTG ATG GTT ATG AAG C	Amplifying *p66* locus	(Ristow et al., 2012)
OLCR12 Rev Comp	CAT TTT AGC AAC AGG ATT GCT	Amplifying *p66* locus	This study
OLCR05	ATT TGC AAG GAA AGA AAT ATA AGG	Sequencing *p66* locus	(Ristow et al., 2012)
OLCR06	TGT TGA AAT GGA TGC TAT TGG	Sequencing *p66* locus	(Ristow et al., 2012)

OLCR07	TTG AAG ATG CAA TGA AAC TCG	Sequencing *p66* locus	(Ristow et al., 2012)
OLCR08	GGG ATT ATA AAT GGA TTA GGA TGG	Sequencing *p66* locus	(Ristow et al., 2012)
OLCR09	TGC ATT TTC AAC AGG AGC AA	Sequencing *p66* locus	(Ristow et al., 2012)
OLCR10	TCA AGA GAA TGA CAA AGA CAC TCC	Sequencing *p66* locus	(Ristow et al., 2012)
OLCR11	TTT CAA ACC CAG GAA CAA GC	Sequencing *p66* locus	(Ristow et al., 2012)
OJLC29	ATA AAG GAT TCC TTG ATA TGT TTT ATT	Sequencing *p66* locus	(Ristow et al., 2012)
OJLC32	CAC TAA AAG CGG AAG GCA AAA AAG GC	Sequencing *p66* locus	(Ristow et al., 2012)
OJLC35	CGC CCA GGA TTC TTT TTC ACC GGT A	Sequencing *p66* locus	(Ristow et al., 2012)
ONN660	AAT ATG GCC TTG AAT TTT TAC CTA ATA	Sequencing *p66* locus	(Ristow et al., 2012)
B garinii 1R	CAG AAT TGT ATA CCA TCT TTC ATT CTG C	Amplifying *p66* locus in *B. garinii;* Sequencing *p66* locus in *B. garinii*	This study
B garinii 2F	GCT TCA TAA CCA TCA GAT TCA AGT TC	Sequencing *p66* locus in *B. garinii*	This study
B garinii 3F	GCA AAT CTT TTC ATA AGC ATC TTG	Sequencing *p66* locus in *B. garinii*	This study
B garinii 3R	CAA GAT GCT TAT GAA AAG ATT TGC	Sequencing *p66* locus in *B. garinii*	This study
B garinii 4F	CTC TTG GCT GGG ATT CAA ATA AC	Sequencing *p66* locus in *B. garinii*	This study
B garinii 4R	GTT ATT TGA ATC CCA GCC AAG AG	Sequencing *p66* locus in *B. garinii*	This study

B garinii 5F	GCA CAC AAG CTA CAA TCC CTA ATC	Sequencing *p66* locus in *B. garinii*	This study
B garinii 5R	GAT TAG GGA TTG TAG CTT GTG TGC	Sequencing *p66* locus in *B. garinii*	This study
B garinii 6F	GTT GAA ATC TCA AGC TAT GAA GAC	Sequencing *p66* locus in *B. garinii*	This study
B garinii 6R	GTC TTC ATA GCT TGA GAT TTC AAC	Amplifying *p66* locus in *B. garinii;* sequencing *p66* locus in *B. garinii*	This study
B garinii 7F	GAG TCC AAC TTT ATC AAA TTC TGC	Amplifying *p66* locus in *B. garinii,* sequencing *p66* locus in *B. garinii*	This study
B garinii 7R	GCA GAA TTT GAT AAA GTT GGA CTC	Sequencing *p66* locus in *B. garinii*	This study
B garinii 8F	GCG CCT ATG ACC GGA TTC AAA AGC	Sequencing *p66* locus in *B. garinii*	This study
B garinii 8R	GCT TTT GAA TCC GGT CAT AGG CGC	Sequencing *p66* locus in *B. garinii*	This study
B garinii 9F	GAC GAT CCA TTC TCA GCT TAC	Sequencing *p66* locus in *B. garinii*	This study
B garinii 9R	GTA AGC TGA GAA TGG ATC GTC	Sequencing *p66* locus in *B. garinii*	This study

B. garinii was amplified in two PCR products (B garinii 1R + B garinii 7F) and (B garinii 6R and OLCR 12 Rev Comp). Sequencing *B. garinii p66* was achieved with the *B. garinii* specific primers and OLCR03, OLCR09, and OLCR10.

2.2 Supplementary Table 2: Minimum inhibitory concentrations to vancomycin (µg/ml)

Strain	Mutation Type	MIC: Median (Range)	# of dilutions from WT
B31 A3 WT	None	.5 (.5-1)	NA
B31 A3 K04 C3-14 (Δ*p66*)	Directed	2 (2-4)	2
B31 A3 P66 E56*	Spontaneous	2 (2-2)	2
B31 A3 P66 Y98*	Spontaneous	2 (2-2)	2
B31 A3 P66 G175V	Spontaneous	4 (2-4)	2
B31 A3 P66 E227*	Spontaneous	2 (2-2)	2
B31 A3 P66 E422*	Spontaneous	2 (2-2)	2
B31 A3 P66 G175V clone A10	SDM	2 (2-2)	2
B31 A3 P66 G175V clone A16	SDM	2 (2-2)	2
B31 A3 P66 G175V clone B11	SDM	2 (2-2)	2
B31 A3 P66 T176M clone A1	SDM	2 (2-2)	2
B31 A3 P66 T176M clone A2	SDM	2 (2-2)	2
B31 A3 P66 T176M clone A6	SDM	2 (2-2)	2
B31 A3 P66 G584R clone A39	SDM	2 (2-2)	2
B31 A3 P66 G584R clone A41	SDM	2 (2-2)	2
B31 A3 P66 G584R clone C39	SDM	2 (2-2)	2
B31 A3 P66 G175V + D299N	°Spontaneous	.5 (.5-.5)	0
B31 A3 P66 G175V + G199E	°Spontaneous	.5 (.5-.5)	0
B31 A3 P66 G175V, Control 1	°None	2 (2-2)	2
B31 A3 P66 G175V, Control 2	°None	2 (2-2)	2
B31 A3 P66 T176M + K41I	°Spontaneous	1 (1-1)	1
B31 A3 P66 T176M + S42I	°Spontaneous	1 (.5-1)	1
B31 A3 P66 T176M, Control 1	°None	2 (1-2)	1 or 2
B31 A3 P66 T176M, Control 2	°None	2 (1-2)	1 or 2
HB19 WT	None	1 (.5-2)	NA

HB19 KO4 3-8B (Δ*p66*)	Directed	2 (1-4)	1
HB19 P66 T176M (clone 1)	Spontaneous	2 (1-4)	1
HB19 P66 T176M (clone 5)	Spontaneous	2 (1-4)	1
HB19 P66 T176M (clone 10)	Spontaneous	2 (1-4)	1
HB19 P66 G585R	Spontaneous	2 (1-4)	1
B. garinii PBi WT	None	.5 (.5-.5)	NA
B. garinii PBi P66 Y99*	Spontaneous	2 (2-2)	2
B. garinii PBi P66 I403*	Spontaneous	2 (2-2)	2
B. garinii PBi P66 Y385*	Spontaneous	2 (2-2)	2

° relates to a secondary mutation

2.3 Supplementary Table 3. Statistical comparison of growth between *B. burgdorferi* strains in BSKII and BSKII + vancomycin for double mutants (in conjunction with Figure 10)

	**BSKII**		**BSKII + vancomycin**	
	**WT**	***Δp66***	**WT**	***Δp66***
WT	-	ns	-	p<0.0001
Δp66	ns	-	p<0.0001	-
P66 G175V	ns	ns	p=0.0065	ns
P66 G175V, Post Inf	ns	ns	p=0.0002	ns
P66 G175V + G199E	ns	ns (n=0.0564)	ns	p<0.0001
P66 G175V + D299N	ns	ns	ns	p<0.0001

	**BSKII**		**BSKII + vancomycin**	
	**WT**	***Δp66***	**WT**	***Δp66***
WT	-	p=0.0146	-	p=0.0002
Δp66	p=0.0146	-	p=0.0002	-
P66 T176M	ns	p=0.0013	p<0.0001	ns
P66 T176M, Post Inf	p=0.0326	ns	p=0.0330	p=0.0177
P66 T176M + K41I	ns	ns (p=0.0629)	ns	p=0.0002
P66 T176M + S42I	ns	ns	ns	p=0.0002

Simple linear regression on GraphPad Prism 9.2.0 was used to calculate statistically significant differences in slope for the different strains and treatment groups.

References

Curtis, M.W., Fierros, C.H., Hahn, B.L., Surdel, M.C., Kessler, J., Anderson, P.N., Vandewalle-Capo, M., Bonde, M., Zhu, J., Bergström, S. and Coburn, J. (2022). Identification of amino acid domains of *Borrelia burgdorferi* P66 that are surface exposed and important for localization, oligomerization, and porin function of the protein. Frontiers in Cellular and Infection Microbiology 12: 991689.

Morrison Tom, B., Ma, Y., Weis John, H. and Weis Janis, J. (1999). Rapid and sensitive quantification of *Borrelia burgdorferi*-infected mouse tissues by continuous fluorescent monitoring of PCR. Journal of Clinical Microbiology 37(4): 987-992.

Ristow, L.C., Bonde, M., Lin, Y.P., Sato, H., Curtis, M., Wesley, E., Hahn, B.L., Fang, J., Wilcox, D.A. and Leong, J.M. (2015). Integrin binding by *Borrelia burgdorferi* P66 facilitates dissemination but is not required for infectivity. Cellular Microbiology 17(7): 1021-1036.

Ristow, L.C., Miller, H.E., Padmore, L.J., Chettri, R., Salzman, N., Caimano, M.J., Rosa, P.A. and Coburn, J. (2012). The β_3-integrin ligand of *Borrelia burgdorferi* is critical for infection of mice but not ticks. Molecular Microbiology 85(6): 1105-1118.
